# Construction of Bimetallic and Trimetallic Aggregates with a [Y(COT)_2_]^−^ Building Block: Solid-State Structures and Solution Behavior

**DOI:** 10.1021/acs.organomet.5c00233

**Published:** 2025-09-08

**Authors:** Zheng Zhou, Aaron J. Babson, Zheng Wei, Marina A. Petrukhina

**Affiliations:** a Department of Chemistry, 1084 University at Albany, State University of New York, Albany, New York 12222, United States; b School of Materials Science and Engineering, 12476 Tongji University, Shanghai 201804, China

## Abstract

Several new bimetallic and trimetallic complexes containing the [Y­(COT)_2_]^−^ unit in different coordination environments are synthesized and fully characterized using X-ray crystallography and NMR spectroscopy. These complexes include [K([2.2.2]­cryptand)]­[Y­(COT)_2_] (**1**), [K­(18-crown-6)­(THF)]­[Y­(COT)_2_] (**2**), [Y_2_K_2_(COT)_4_(THF)_4_] (**3**), and [YKCa­(COT)_3_(THF)_3_] (**4**). In the presence of secondary ligands, bimetallic ionic pairs that comprise a [Y­(COT)_2_]^−^ sandwich and a solvent-wrapped K^+^ ion are formed in **1** and **2**. In contrast, **3** represents a bimetallic tetradecker oligomer with two [Y­(COT)_2_]^−^ units bridged by a K^+^ ion and a terminal [K­(THF)_4_]^+^ moiety. Complex **4** is a hetero*tri*metallic triple-decker with an axial arrangement of three metal centers to form [Y­(COT)_2_K­(COT)­Ca­(THF)_3_]. Structural analysis shows the Y–COT_centroid_ distances in the [Y­(COT)_2_]^−^ sandwich are equidistant (1.884(6) Å) in **1** but become asymmetric in **2**–**4** (1.857(3)–1.952(3) Å), reflecting additional external coordination. The two COT rings are parallel in **1** and become increasingly tilted from 0.8 to 27.5° in **2**–**4**, respectively. Multinuclear NMR spectroscopy measurements reveal the solution behavior of the sandwich and heterometallic multidecker COT-based products. The observed NMR spectroscopic trends corroborate with weak interactions between the [Y­(COT)_2_]^−^ unit and cationic moieties persisting in solutions of **2**–**4.**

## Graphical abstract

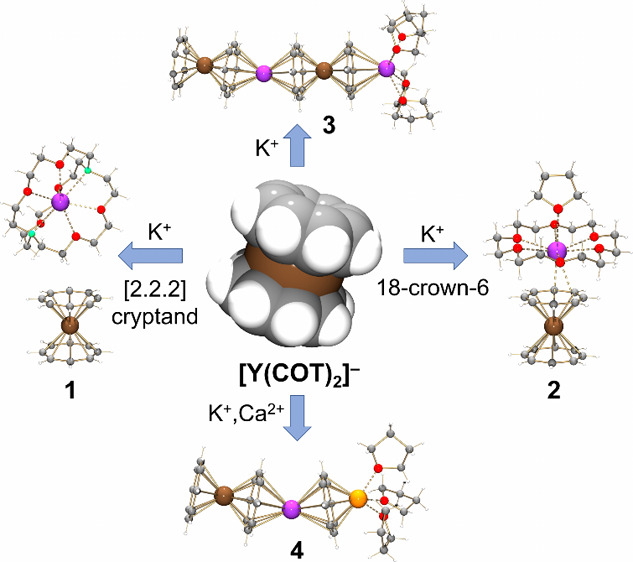

## Introduction

The use of yttrium­(III) compounds as catalysts has seen increased demand in various organic processes, including polymerization, silylation, and hydroamination.^1–5^ In addition to Lewis acid catalysis via YCl_3_, applications utilizing yttrium are found in medical imaging and radiotherapy.^6^ On the other hand, cyclooctatetraene (COT) plays an important role in d- and f-element chemistry, as its aromatic doubly reduced planar cyclooctatetraenyl anion can host a wide range of ionic radii, ranging over 1.380–0.605 Å.^7,8^ Widespread use of COT^2–^ ligands enabled crystallographic characterization of various lanthanide (Ln) COT-based products, with single molecule magnet (SMM) and photoluminescent properties.^9–11^ In these studies of Ln-COT complexes, the Y-analogs are often used to dilute SMMs and to quench quantum tunneling phenomena.^12–14^ In 2013, the dilution of [K­(18-crown-6)]^+^[Er­(COT)_2_]^−^ with isomorphous [K­(18-crown-6)]^+^[Y­(COT)_2_]^−^ in a 1:85 ratio showed a 1.5 times greater coercive field, compared to the neat Er­(III) complex.^14^ Crystallographic study of [K­(18-crown-6)]^+^[Y­(COT)_2_]^−^ revealed two unique [Y­(COT)_2_]^−^ units with dihedral angles of 0.0(1) and 1.6(1)°; however, the product lacked solution behavior characterization.^14^

In addition to ^1^H and ^13^C NMR spectroscopy, ^89^Y NMR provides a good tool for characterization of organometallic Y­(III) products in solutions. The sensitivity of the naturally abundant ^89^Y isotope enables narrow peaks in ^89^Y NMR spectra, allowing reliable comparison of its coordination environment.^11,15,16^ The ^89^Y NMR spectra of Y-COT complexes display a large range of chemical shifts based on the number and coordination mode of COT^2–^. When coordinated to one COT^2–^ or 1,4-trimethylsilane COT (COT^1,4‑TMS^), ^89^Y chemical shifts range from 61.8 to 73.6 ppm.^11,17–19^ However, when sandwiched between two COT or COT^1,4‑TMS^, the chemical shifts exhibit a significant upfield shift, ranging from – 195.5 to – 209.7 ppm.^20^ In heteroleptic sandwiches containing COT and the aromatic ligand, (Cp^ttt^)^−^ (Cp^ttt^ = 1,2,4-tri­(*tert*-butyl)­cyclopentadienide), the observed ^89^Y shift is downfield from the homoleptic sandwiches, with a value of – 149.16 ppm.^15^ Overall, the coordination environment of Y­(III) provides specific ^89^Y shifts to reliably evaluate the solution behavior of the COT-based complexes. To the best of our knowledge, only the above reports demonstrated the great potential of the ^89^Y NMR spectroscopy for the characterization of Y-COT products.

In this work, we used the [Y­(COT)_2_]^−^ building block for constructing various heterometallic complexes ranging from the sandwich-type assemblies to tri- and tetra-decker oligomers. As a result, several new bimetallic and trimetallic complexes with different external coordination of the [Y­(COT)_2_]^−^ unit were synthesized and fully characterized by X-ray diffraction methods. This was followed by investigation of their solution behavior using multinuclear ^1^H, ^13^C, ^89^Y, and DOSY NMR spectroscopy.

## Results and Discussion

The preparation of new organometallic yttrium­(III) cyclooctatetraenyl complexes has been achieved by a facile one-pot synthesis (Scheme 1). For compounds **1**–**3**, the solution of K_2_COT (2 equiv) in THF was added dropwise to the solution of YCl_3_ in THF at room temperature, and the reactions were allowed to proceed to completion over 24 h. The products were crystallized as yellow blocks or plate-shaped crystals in good yield using slow diffusion of hexanes (in the presence of [2.2.2]­cryptand or 18-crown-6) to the THF filtrate. For compound **4**, the solution of K_2_COT (3 equiv) was added dropwise to an equimolar mixture of YCl_3_ and CaI_2_ in THF at room temperature, and the mixture was stirred for 24 h. After removing the KI and KCl salts by filtration, the product was crystallized using the same diffusion method to afford yellow plate-shaped crystals. The X-ray diffraction study confirmed the formation of the following complexes, namely [K([2.2.2]­cryptand)]­[Y­(COT)_2_] (**1**), [K­(18-crown-6)­(THF)]­[Y­(COT)_2_] (**2**, crystallized with one interstitial THF molecule as **2**·THF), [Y_2_K_2_(COT)_4_(THF)_4_] (**3**), and [YKCa­(COT)_3_(THF)_3_] (**4**).

**Scheme 1:**
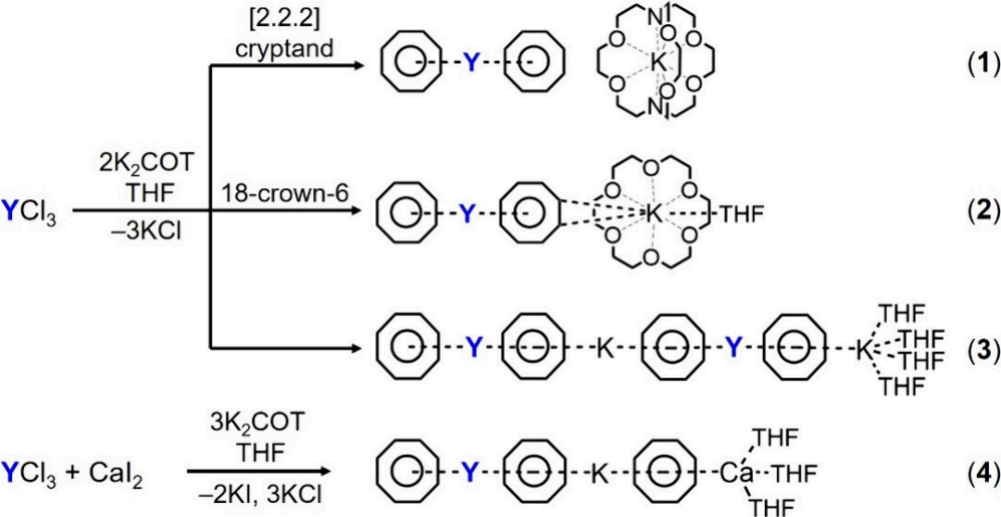
Synthesis of Complexes **1**–**4.**

In the crystal structure of **1** (Figure 1a), the {K^+^[2.2.2]­cryptand} cationic moiety is separated from the anionic core to afford a “naked” [Y­(COT)_2_]^−^ sandwich. The Y­(III) ion is symmetrically placed between two parallel COT^2–^ ligands (180°) with a η^8^-coordination mode (Y–COT_centroid_: 1.884(6) Å), consistent with its Ln analogs (Ce, Pr, Nd, and Sm).^21^ The K^+^ ion is fully wrapped by a [2.2.2]­cryptand molecule with the K–O (2.799(4)–2.857(4) Å) and K–N (2.957(5) Å) distances being close to the previously reported values.^21–23^ Although the “naked” [Y­(COT)_2_]^−^ sandwich has been previously observed in [K­(18-crown-6)­(THF)_2_]­[Y­(COT)_2_]^14^ and [Li­(DME)_3_]­[Y­(COT″)_2_] (where COT″ = 1,4-bis­(trimethylsilyl)­cyclooctatetraenyl dianion),^24^ the two COT^2–^ decks in the reported structures were tilted (1.6° and 3.8°, respectively) with a slight variation of the Y–COT distances (1.888(7)/1.899(7) Å and 1.891(2)/1.907(2) Å, respectively). The same product composition is also observed in two crystallographically characterized complexes of Gd^25^ and Er.^26^ The Er­(III) complex is isomorphous to **1**, while the arrangement and coordination environment of [Gd­(COT)_2_]^−^ sandwiches in the solid state is different from that of **1.**

**Figure 1:**
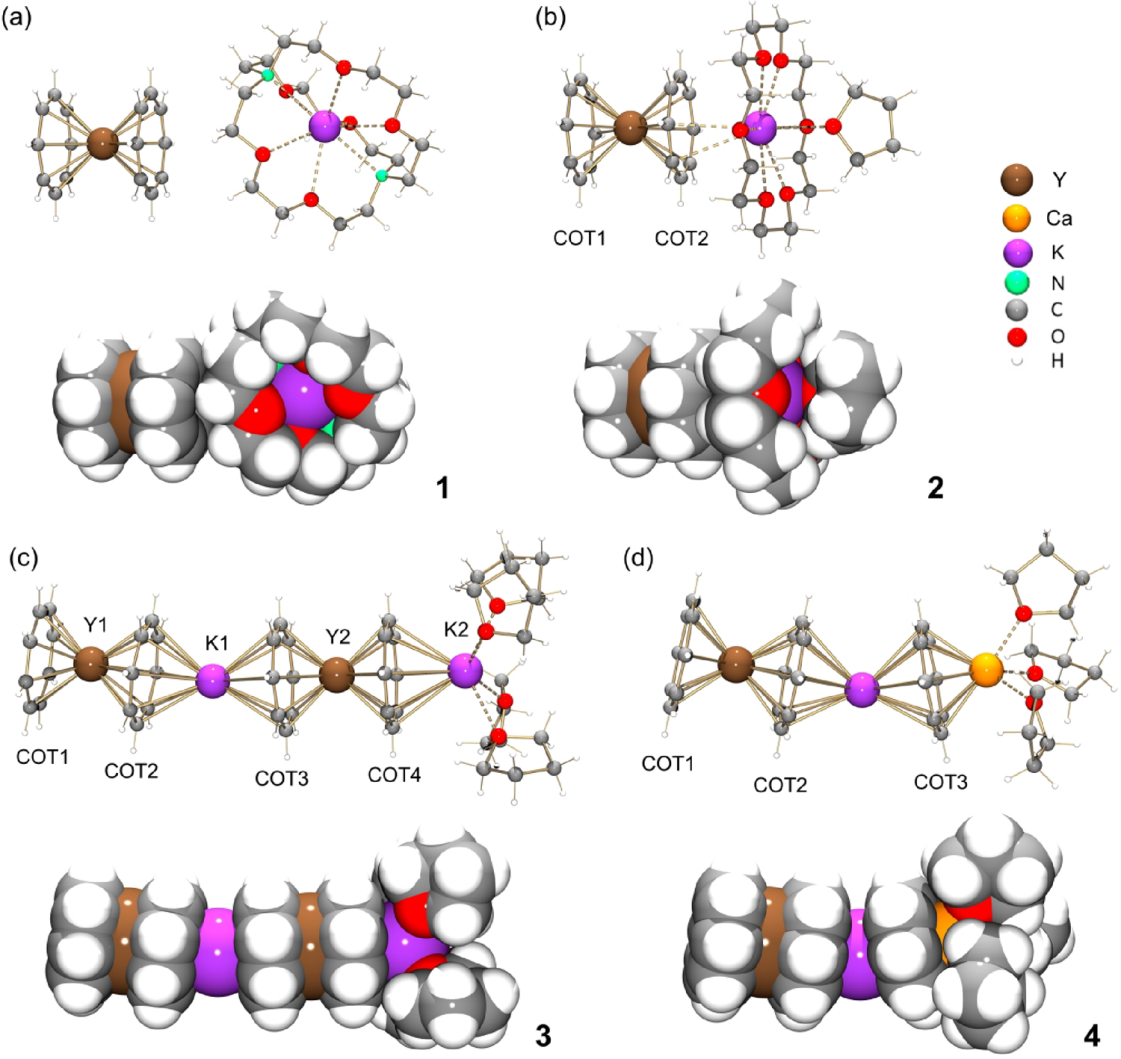
Crystal structures of (a) [K([2.2.2]­cryptand)]­[Y­(COT)_2_] (**1**), (b) [K­(18-crown-6)­(THF)]­[Y­(COT)_2_] (**2**), (c) [Y_2_K_2_(COT)_4_(THF)_4_] (**3**), and (d) [YKCa­(COT)_3_(THF)_3_] (**4**), ball-and-stick and space-filling models.

In the crystal structure of **2** (Figure 1b), the Y­(III) ion is sandwiched by two η^8^-coordinated COT^2–^ ligands (COT1 and COT2) with the Y–COT_centroid_ distances of 1.883(4) Å and 1.892(4) Å and a small tilting angle of 2.2°, that is also observed in the isostructural products containing Dy and Er.^7,27,28^ The K^+^ ion is bound to the edge of COT2 in a η^2^-fashion (K–C: 3.231(2)/3.310(4) Å). It is also trapped by an 18-crown-6 ether (K–O_crown_: 2.776(2)–2.894(2) Å) and caped with one THF molecule (K–O_THF_: 3.034(3) Å).

In the crystal structure of the tetranuclear complex **3** (Figure 1c), both Y­(III) ions are sandwiched by two η^8^-coordinated COT^2–^ anions, and the two sandwiches are bridged by one K^+^ ion with a terminal {K^+^(THF)_4_} moiety. The resulting bimetallic tetradecker oligomeric structure is analogous to the complexes reported with several lanthanides, such as Gd, Er, and Tm.^27–29^ The tilting angles of two [Y­(COT)]^−^ sandwiches are 3.0° and 2.2°, with the values close to those in the Ln–K–Ln–K tetramers.^20–22^ Notably, the Y1–COT_centroid_ distances of 1.857(3) Å and 1.952(3) Å show that Y1 sits closer to COT1 than to COT2 (Δ = 0.11 Å), which can be attributed to the engagement of the COT2 ring in the external K^+^ ion binding. In contrast, the Y2 ion placement between two COT rings is more symmetric with the Y2–COT_centroid_ distances of 1.897(3) Å and 1.883(3) Å, respectively. The bridging K^+^ ion is also sandwiched by two η^8^-coordinated COT^2–^ ligands with the K–C distances of 3.069(3)–3.129(3) Å and the tilting angle of 11.6°. The second K^+^ ion is terminally bound to COT4 in a η^8^-fashion (K–C: 3.105(3)–3.267(3) Å) and capped by four THF molecules (K–O_THF_: 2.695(2)–2.708(2) Å).

In the crystal structure of hetero*tri*metallic complex **4** (Figure 1d), the Y­(III) ion is sandwiched by two η^8^-coordinated COT^2–^ ligands, with the two rings nearly parallel (0.8°). This small deviation is commonly seen in the substituted COT sandwiches.^9,27,28,30^ The Y–COT_centroid_ distances are 1.859(2) Å and 1.907(2) Å. The asymmetry observed in **4** (Δ ≈ 0.04 Å) is slightly larger than in most [Y­(COT)_2_]^−^ sandwiches, but is smaller than in the [Y­(COT)_2_]^−^ sandwich in **3** and the previously reported triple-decker [Y_2_(COT)_3_].^27^ The central K^+^ ion is also sandwiched by two η^8^-coordinated COT^2–^ ligands (K–C: 2.977(8)–3.118(8) Å) but with a noticeably larger tilting angle of 27.5°. The Ca^2+^ ion terminates the complex by an η^8^-coordination to the COT4, with the Ca–C distances of 2.648(2)–2.677(2) Å. The coordination environment of Ca^2+^ is fulfilled by three capping THF molecules (Ca–O_THF_: 2.371(1)–2.401(1) Å). The Y–K–Ca angle of 153.6° shows a greater deviation from linearity in the trimetallic complex in comparison to **3** (169.4° for Ln–K–Ln and 179.1° for K–Ln–K), but it is consistent with the trend observed for the Ln–K–Ca (Ln = Gd–Yb) series (153.5°–153.6°).^31^

In the solid-state structure of **1** (Figure 2a), multiple interactions are found between the [Y­(COT)]^−^ sandwiches and the {K^+^[2.2.2]­cryptand} moieties, with the shortest C–H···π contacts ranging over 2.668(8)–2.822(8) Å. In contrast, no noticeable interactions are found between adjacent molecules in **2**. In the solid-state structure of **3** (Figure 2c), the unidirectional 1D columns are formed between the open-ended COT1 decks and coordinated THF molecules from the neighboring molecules, with some weak C–H···π interactions (2.863(5) Å). In **4**, the 1D columns are also formed between the open-ended COT1 decks and coordinated THF molecules from neighboring molecules (C–H···π interactions: 2.708(5)–2.751(5) Å), but these columns are oriented in opposite directions (Figure 2d). No significant interactions are found between the adjacent columns in **3** and **4.**

**Figure 2:**
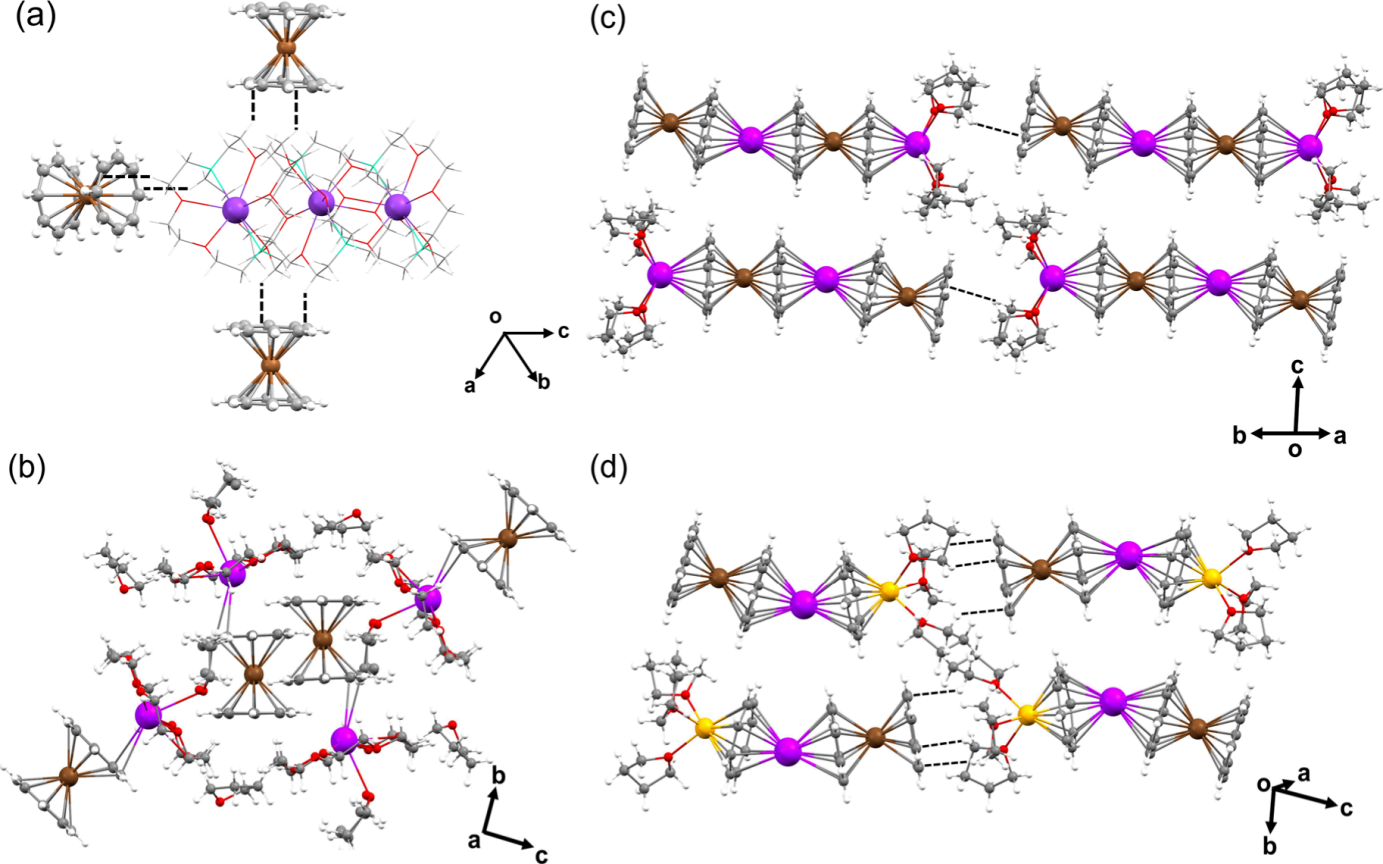
Solid-state packing of **1** (a) mixed model, **2** (b), **3** (c), and **4** (d), ball-and-stick models. C–H···π contacts are shown as dashed lines.

### NMR Spectroscopic Characterization

To gain insights into the solution behavior of the heterometallic complexes having [Y­(COT)_2_]^−^ units in different coordination environments, the ^1^H NMR investigation was carried out for **1**–**4**. In the ^1^H NMR spectrum of **1** (Figure 3), the singlet of COT^2–^ at 5.69 ppm corresponds to the “naked” [Y­(COT)_2_]^−^ sandwich. In the ^1^H NMR spectrum of **2**, the proton signal of COT^2–^ is shifted slightly downfield to 5.75 ppm, which may stem from the weak interaction persisting between the [Y­(COT)_2_]^−^ sandwich and the {K^+^(18-crown-6)} moiety in solution. These data are consistent with the previously reported diamagnetic sandwich complexes containing COT^2–^ which display a range of chemical shifts from 5.81 to 6.52 ppm in the ^1^H NMR spectra.^10,15,30,32,33^

**Figure 3:**
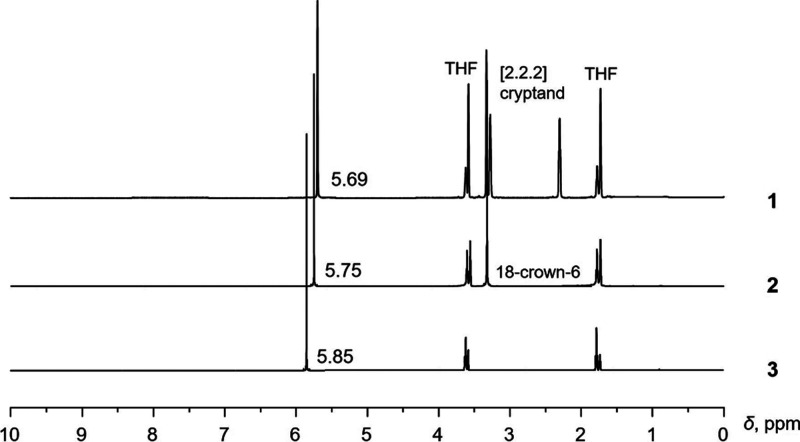
^1^H NMR spectra of **1**–**3** in THF-*d*_*8*_ at 25 °C.

Notably, the proton signal of COT^2–^ in **3** also appears as a singlet, indicating the tetramer’s dissociation into two similar subunits in THF. Compared to **2**, the more deshielded proton signal of COT^2–^ in **3** (5.85 ppm) could result from stronger coordination of K^+^ ion to the [Y­(COT)_2_]^−^ sandwich in the absence of such coordinating agents as crown ether or cryptand in this case. Overall, it can be concluded that enhanced interactions from {K^+^[2.2.2]­cryptand} to {K^+^(18-crown-6)} lead to the downfield shift of proton signal of COT^2–^. Moreover, the presence of several [Y­(COT)_2_]^−^ units in a row in the oligomeric structure would further enhance the deshielding effect.

In the ^1^H NMR spectra of hetero*tri*metallic complex **4** (Figure 4), there are two COT^2–^ signals at 5.74 and 6.04 ppm with an integration ratio of 2:1 at room temperature. Those signals become better resolved and slightly shifted to 5.70 and 6.02 ppm at – 80 °C. In our previously reported [Y_2_(COT)_3_(THF)_2_] complex,^30^ the proton signal of COT^2–^ at 5.92 ppm was found to split into two singlets upon cooling (5.88 and 6.28 ppm), revealing a relatively strong interaction between the [Y­(COT)_2_]^−^ and [Y­(COT)­(THF)_2_]^+^ fragments that persists in solution. In contrast, the chemical shift of [Y­(COT)_2_]^−^ fragment in **4** (5.74 ppm) is smaller than those in **2** and **3** and is further shielded at low temperature, with a value being close to a “naked” sandwich (**1**). Therefore, it is very likely that the heterotrimetallic structure dissociates into two relatively stable fragments ([Y­(COT)_2_]^−^ and [KCa­(COT)­(THF)_3_]^+^) in THF solution.

**Figure 4:**
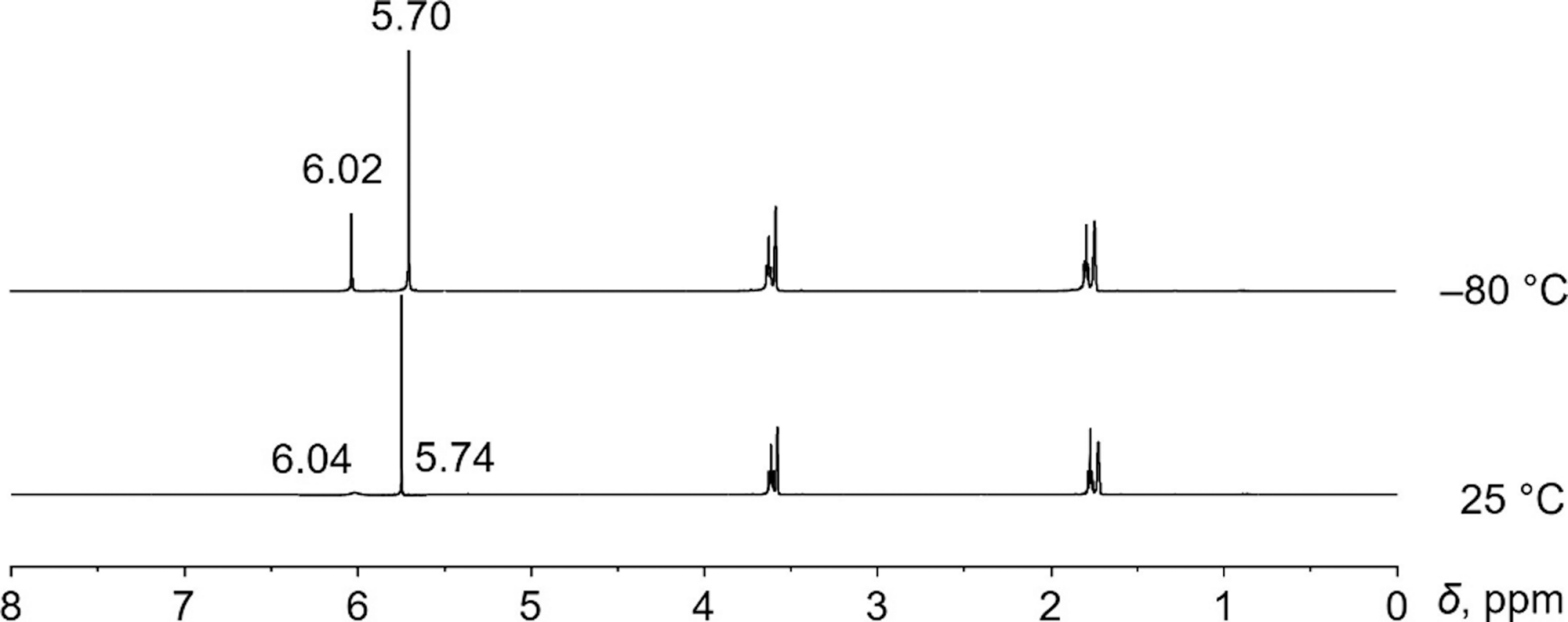
^1^H NMR spectra of **4** in THF-*d*_*8*_ at 25 °C and – 80 °C.

To confirm the dynamic solution behavior of compounds **3** and **4**, both solid-state ^13^C NMR, and Diffusion Ordered Spectroscopy (DOSY) NMR characterization were completed. The ^13^C Cross-Polarization Magic Angle Spinning (CPMAS) NMR spectra (Figure S21) show significant broadening of the signals of COT^2–^ and complexed THF molecules, as reveled by comparison of multidecker **3** and **4** with double-deckers **1** and **2**. In contrast to the latter, the broad COT^2–^ signal in **3** ranges over 97.2–90.4 ppm, while the coordinated THF molecules show as multiplets in the solid-state spectra. In **4**, the ^13^C signal of COT^2–^ shows further broadening over 97.9–87.6 ppm range, reflecting on the notably different coordination environments of the COT rings in the solid-state structure. In addition, the DOSY spectrum of **3** (Figure S22) revealed the presence of only one fragment in solution; however, two distinct species are present in **4** (Figure S23), consistent with the above assignments.

It should be noted that ^89^Y NMR data show sensitivity to variations in the secondary coordination environment of the [Y­(COT)_2_]^−^ unit in complexes **1**–**4**. To demonstrate these variations for the [Y­(COT)_2_]^−^ sandwich, the ^1^H–^89^Y HMQC spectra (Figures S17–S20) were recorded (Figure 5). In the “naked” [Y­(COT)_2_]^−^ sandwich of **1**, the ^89^Y signal occurs at – 200 ppm; similar to the previously reported solvent-separated [K([2.2.2]­cryptand)]^+^[Y­(dbCOT)_2_]^−^ (where dbCOT = dibenzo-cyclooctatetraene) with a ^89^Y NMR peak at – 202 ppm.^16^ The signals in **2** and **4** are shifted slightly to – 198 and – 197 ppm, respectively, and further deshielded to – 196 ppm in **3**. This gradual downfield shift is a result of increasing intermolecular interactions between the [Y­(COT)_2_]^−^ unit and cationic moieties in **1**–**3**, which is consistent with the trends observed in ^1^H NMR spectra (Figure 3). These trends are also consistent with structural variations observed in the series (Table S7). Interestingly, the ^89^Y signal in **4** found between those in **2** and **3** could be indicative of weak interaction persisting between the [Y­(COT)_2_]^−^ sandwich and the [KCa­(COT)­(THF)_3_]^+^ moiety in solution.

**Figure 5:**
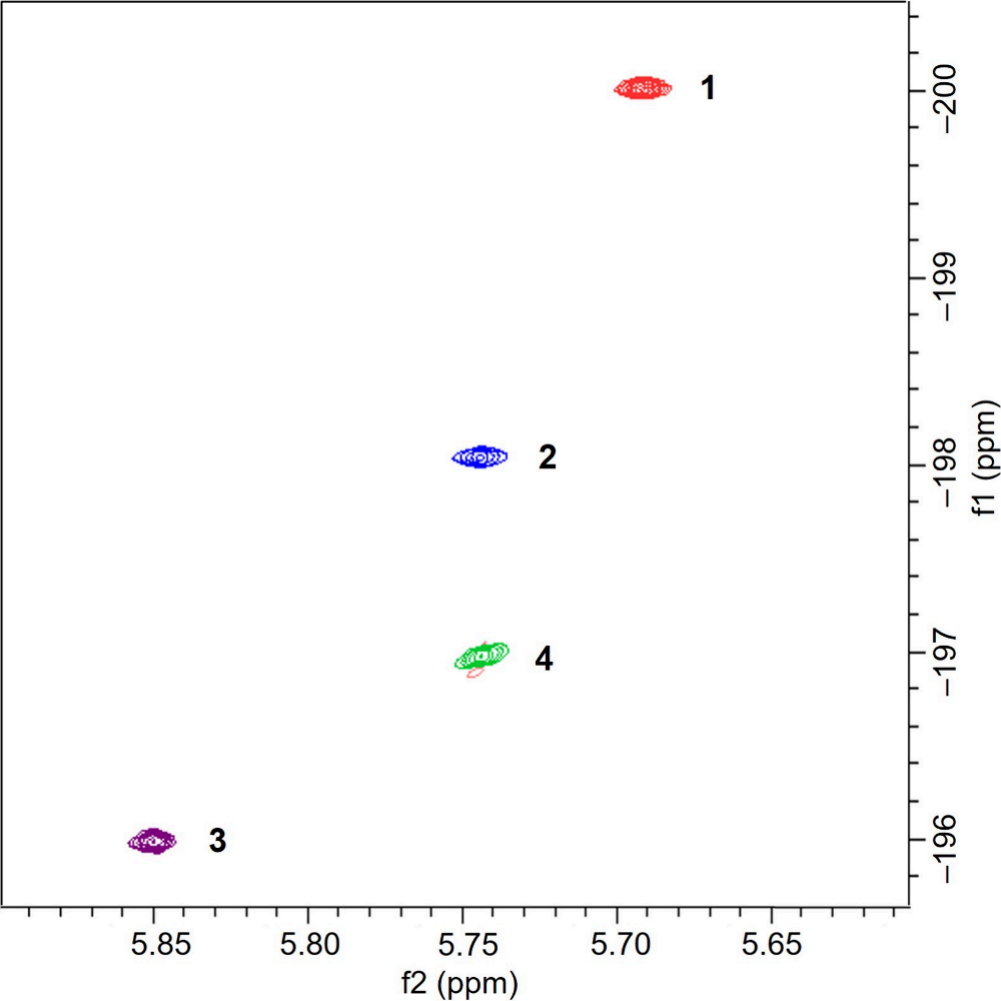
Comparison of ^1^H–^89^Y HMQC spectra of **1**–**4** in THF-*d*_8_ at 25 °C.

## Conclusion

A facile and effective one-pot synthetic route for preparing different types of yttrium­(III) cyclooctatetraenyl-based complexes has been developed. Several new bimetallic and trimetallic complexes containing the [Y­(COT)_2_]^−^ building unit have been successfully synthesized and fully characterized using X-ray diffraction and multinuclear NMR spectroscopy. In the presence of secondary ligands, bimetallic products comprised of a [Y­(COT)_2_]^−^ sandwich and a cationic moiety are formed as [K([2.2.2]­cryptand)]­[Y­(COT)_2_] and [K­(18-crown-6)­(THF)]­[Y­(COT)_2_]. In contrast, the bimetallic tetradecker, [Y_2_K_2_(COT)_4_(THF)_4_], represents an oligomer with two [Y­(COT)_2_]^−^ units bridged by a K^+^ ion and terminated by a [K­(THF)_4_]^+^ moiety. Moreover, the first hetero*tri*metallic complex containing Y^3+^ exhibits a triple-decker structure with an axial arrangement of three different metal centers in [YKCa­(COT)_3_(THF)_3_]. The detailed structural analysis shows that the symmetry of the [Y­(COT)_2_]^−^ sandwich is affected by its external coordination environment, with the Y–CO*T*_centroid_ distances ranging from 1.857(3) to 1.952(3) Å. Weak interactions between the [Y­(COT)_2_]^−^ unit and external cationic moieties are responsible for dissociation/binding pathways detected in solutions with the help of ^1^H and ^89^Y NMR spectroscopic studies. Overall, the observed NMR spectroscopic trends are consistent with variations in coordination environments of the [Y­(COT)_2_]^−^ unit revealed by X-ray crystallography.

## Experimental Section

### Materials and Methods

All manipulations were carried out using break-and-seal and glovebox techniques^34,35^ under an atmosphere of argon. Tetrahydrofuran (THF) and hexanes (Sigma-Aldrich) were dried over Na/benzophenone and distilled prior to use. THF-*d*_8_ (Sigma-Aldrich) was dried over NaK_2_ alloy and vacuum-transferred. Cyclooctatetraene (COT, 98%, Sigma-Aldrich), potassium (98%, Sigma-Aldrich), 18-crown-6 ether (99%, Sigma-Aldrich), [2.2.2]­cryptand (99.0%, Sigma-Aldrich), CaI_2_ (99.95%, Sigma-Aldrich), and YCl_3_ (99.9%, Strem Chemicals) were used as received. K_2_COT was prepared as described previously.^30^ Attenuated total reflection (ATR) IR spectra were recorded on a PerkinElmer Spectrum 100 FT-IR spectrometer. ^1^H NMR spectra were measured using a Bruker Ascend-500 spectrometer (500 MHz for ^1^H) with a 5 mm BBO probe and referenced to the resonances of the solvent used. The low-temperature NMR experiment was controlled by a Cryo Diffusion cryogenic tank probe, and liquid N_2_ was used as a cooling source. ^89^Y and ^13^C NMR spectra were measured using an Avance 400 MHz spectrometer (19.6 MHz for ^89^Y and 100 MHz for ^13^C) with an iProbe BBFO probe and referenced to 1 M of YCl_3_ D_2_O. Solid state NMR spectra were collected using a JEOL JNM 600R spectrometer equipped with an Automated Solid State NMR System probe referenced to adamantane at 37.777 ppm. Crystalline samples **1**–**4** were packed into 49 μL, 3.2 mm zirconia rotors inside an argon atmosphere glovebox. ^13^C Cross-Polarization Magic Angle Spinning (CPMAS) NMR spectra were collected with a MAS frequency of 18 kHz at 25 °C. The DOSY NMR spectra were collected on a JEOL JNM 600R spectrometer using 64 scans with a diffusion time of 100 ms. A magnetic field gradient of 5 mT/m was used with a delay time of 50 ms. The data were processed using Delta v6.4 and JASON v5.2 for diffusion coefficient calculations. X-ray powder diffraction data were collected on a Bruker D8 VENTURE diffractometer (Cu INCOATEC IμS microfocus X-ray Kα radiation, mirror monochromator, PHOTON 100 CMOS area detector, – 173.15 °C). The crystalline samples under investigation were ground under Ar in the glovebox and mounted on the MiTeGen dual thickness MicroMounts sample holder (30 μm) or placed in the dome-like airtight zero background holders. Le Bail fit for powder diffraction patterns was performed using TOPAS, version 4 software package (Bruker AXS, 2006).

#### [K­([2.2.2]­cryptand)]­[Y­(COT)_2_] (1)

YCl_3_ (20 mg, 0.10 mmol) was stirred in THF (2.0 mL) under an argon atmosphere at 25 °C for 4 h. Slow addition of K_2_COT (36 mg, 0.20 mmol, in 1.0 mL of THF) to a rapidly stirring solution produced a cloudy yellow suspension with a large amount of white precipitate that formed in 30 min. The mixture was allowed to stir under an argon atmosphere at 25 °C for 24 h to complete the reaction. Then it was filtered, and the yellow filtrate was layered with 1.5 mL of anhydrous hexanes containing [2.2.2]­cryptand (104 mg, 0.28 mmol). The ampule was sealed under argon and stored at 5 °C. Yellow blocks were present in good yield after 5 days. Yield: 53 mg, 75%. ATR-IR: 678, 741, 781, 891 cm^–1^. ^1^H NMR (500 MHz, THF-*d*_8_, 25 °C, ppm): δ 5.69 (s, 16H, C_8_H_8_^2–^), 3.30 (s, 12H, [2.2.2]­cryptand), 3.27 (d, J = 4.9 Hz, 12H, [2.2.2]­cryptand). 2.30 (d, J = 5.9 Hz, 12H, [2.2.2]­cryptand). ^13^C NMR (126 MHz, THF-*d*_8_, 25 °C, ppm): δ 94.1 (C_8_H_8_^2–^). ^89^Y NMR (19.6 MHz, THF-*d*_8_, 25 °C, ppm): δ – 200 ([Y­(C_8_H_8_^2–^)_2_]^−^).

#### [K­(18-crown-6)­(THF)]­[Y­(COT)_2_] (2)

YCl_3_ (20 mg, 0.10 mmol) was stirred in THF (2.0 mL) under an argon atmosphere at 25 °C for 4 h. Slow addition of K_2_COT (36 mg, 0.20 mmol, in 1.0 mL of THF) to a rapidly stirring solution produced a cloudy yellow suspension with a large amount of white precipitate that formed in 30 min. The mixture was allowed to stir under an argon atmosphere at 25 °C for 24 h to complete the reaction. Then it was filtered, and the yellow filtrate was layered with 1.5 mL of anhydrous hexanes containing 18-crown-6 (26 mg, 0.28 mmol). The ampule was sealed under argon and stored at 5 °C. Yellow blocks were present in good yield after 5 days. Yield: 59 mg, 80%. ATR-IR: 677, 743, 755, 838, 897, 961, 1056, 1104 cm^–1^. ^1^H NMR (500 MHz, THF-*d*_8_, 25 °C, ppm): δ 5.75 (s, 16H, C_8_H_8_^2–^), 3.62 (m, 16H, THF), 3.32 (s, 24H, 18-crown-6), 1.78 (m, 16H, THF). ^13^C NMR (126 MHz, THF-*d*_8_, 25 °C, ppm): δ 94.1 (C_8_H_8_^2–^). ^89^Y NMR (19.6 MHz, THF-*d*_8_, 25 °C, ppm): δ – 198 ([Y­(C_8_H_8_^2–^)_2_]^−^).

#### [Y_2_K_2_(COT)_4_(THF)_4_] (3)

YCl_3_ (20 mg, 0.10 mmol) was stirred in THF (2.0 mL) under an argon atmosphere at 25 °C for 4 h. Slow addition of K_2_COT (36 mg, 0.20 mmol, in 1.0 mL of THF) to a rapidly stirring solution produced a cloudy yellow suspension with a large amount of white precipitate that formed in 30 min. The mixture was allowed to stir under an argon atmosphere at 25 °C for 24 h to complete the reaction. Then it was filtered, and the yellow filtrate was layered with 1.5 mL of anhydrous hexanes. The ampule was sealed under argon and stored at 5 °C. Yellow plates were present in good yield after 5 days. Yield: 38 mg, 80%. ATR-IR: 664, 680, 707, 741, 779, 882, 893, 1046, 1068 cm^–1^. ^1^H NMR (500 MHz, THF-*d*_8_, 25 °C, ppm): δ 5.85 (s, 32H, C_8_H_8_^2–^), 3.62 (m, 16H, THF), 1.78 (m, 16H, THF). ^13^C NMR (126 MHz, THF-*d*_8_, 25 °C, ppm): δ 93.7 (C_8_H_8_^2–^). ^89^Y NMR (19.6 MHz, THF-*d*_8_, 25 °C, ppm): δ – 196 ([Y­(C_8_H_8_^2–^)_2_]^−^).

#### [YKCa­(COT)_3_(THF)_3_] (4)

CaI_2_ (16 mg, 0.06 mmol) and YCl_3_ (12 mg, 0.06 mmol) were stirred in THF (3.0 mL) under an argon atmosphere at 25 °C for 24 h. Slow addition of K_2_COT (30 mg, 0.17 mmol, in 1.0 mL of THF) to a rapidly stirring solution produced a cloudy yellow suspension with a large amount of white precipitate that formed in 30 min. The mixture was allowed to stir under an argon atmosphere at 25 °C for 48 h to complete the reaction. Then it was filtered, and the yellow filtrate was layered with 2.4 mL of anhydrous hexanes. The ampule was sealed under argon and stored at 5 °C. Yellow blocks were present in good yield after 6 days. Yield: 27 mg, 70%. ATR-IR: 679, 875, 888, 1030 cm^–1^. ^1^H NMR (500 MHz, THF-*d*_8_): δ 6.04 (s, 8H, C_8_H_8_^2–^), 5.74 (s, 16H, C_8_H_8_^2–^), 3.62 (m, 12H, THF), 1.78 (m, 12H, THF) ^1^H NMR – 80 °C (500 MHz, THF-*d*_8_): δ 6.04 (s, 8H, C_8_H_8_^2–^), 5.79 (s, 16H, C_8_H_8_^2–^), 3.62 (m, 12H, THF), 1.79 (m, 12H, THF). ^13^C NMR (126 MHz, THF-*d*_8_): δ 90.1 (C_8_H_8_^2–^), 93.8 (C_8_H_8_^2–^). ^89^Y NMR (19.6 MHz, THF-*d*_8_, 25 °C, ppm): δ – 197 ([Y­(C_8_H_8_^2–^)_2_]^−^).

**X-ray Crystallographic Details** can be found in the Supporting Information file. Deposition numbers 2450813 (**1**), 2450814 (**2**), 2450815 (**3**), and 2450816 (**4**) contain the supplementary crystallographic data for this paper. These data are provided free of charge by the joint Cambridge Crystallographic Data Centre and Fachinformationszentrum Karlsruhe Access Structures service.

## Supplementary Material





## Notes

ATR-IR Spectroscopy, X-ray Powder Diffraction, Crystal Structure Solution and Refinement, ^1^H NMR, ^13^C NMR, ^13^C Solid-State NMR, ^89^Y NMR, and DOSY NMR Spectroscopy, and TD-DFT calculations (PDF)
Text file of all computed molecule Cartesian coordinates in a format for convenient visualization (XYZ)
