# Supplementary material for: Construction of Bimetallic and Trimetallic Aggregates with a [Y(COT)2]− Building Block: Solid-State Structures and Solution Behavior
Source: Organometallics. 2025 Sep 8;44(18):2092–8. doi: 10.1021/acs.organomet.5c00233 (PMC12458972; doi:10.1021/acs.organomet.5c00233)
Supplement: Supplementary file 1 [file om5c00233_si_001.pdf]

Supporting Information

Construction of Bimetallic and Trimetallic Aggregates with a $[Y(COT)_2]^-$ Building Block: Solid-State Structures and Solution Behavior

Zheng Zhou,[a,b] Aaron J. Babson,[a] Zheng Wei,[a] Marina A. Petrukhina[a,*]

[a] *Department of Chemistry, University at Albany, State University of New York, Albany, NY 12222, USA*

[b] *School of Materials Science and Engineering, Tongji University, Shanghai 201804, China*

*mpetrukhina@albany.edu

Supporting Information

I. ATR-IR Spectroscopic Investigation 2

II. X-Ray Powder Diffraction and Le Bail Fit 5

III. ^{1}H NMR Spectroscopic Investigation 9

IV. ^{13}C NMR Spectroscopic Investigation 11

V. ^{89}Y NMR Spectroscopic Investigation 13

VI. ^{13}C Solid-State NMR Spectroscopic Investigation 15

VII. Diffusion Ordered Spectroscopy (DOSY) NMR 16

VIII. Crystal Structure Solution and Refinement Details 18

IX. Calculation Details 25

X. References 29

List of Compounds and Characterization Methods

No.	Formula	CCDC No.	Characterization
**1**	[K([2.2.2]cryptand)][Y(COT)$_2$]	2450813	X-ray, IR, ^{1}H, ^{13}C, ^{89}Y, and solid-state NMR
**2**	[K(18-crown-6)(THF)][Y(COT)$_2$]	2450814	X-ray, IR, ^{1}H, ^{13}C, ^{89}Y, and solid-state NMR
**3**	[Y$_2$K$_2$(COT)$_4$(THF)$_4$]	2450815	X-ray, IR, ^{1}H, ^{13}C, ^{89}Y, DOSY, and solid-state NMR
**4**	[YKCa(COT)$_3$(THF)$_3$]	2450816	X-ray, IR, ^{1}H, ^{13}C, ^{89}Y, DOSY, and solid-state NMR

I. ATR-IR Spectroscopic Investigation

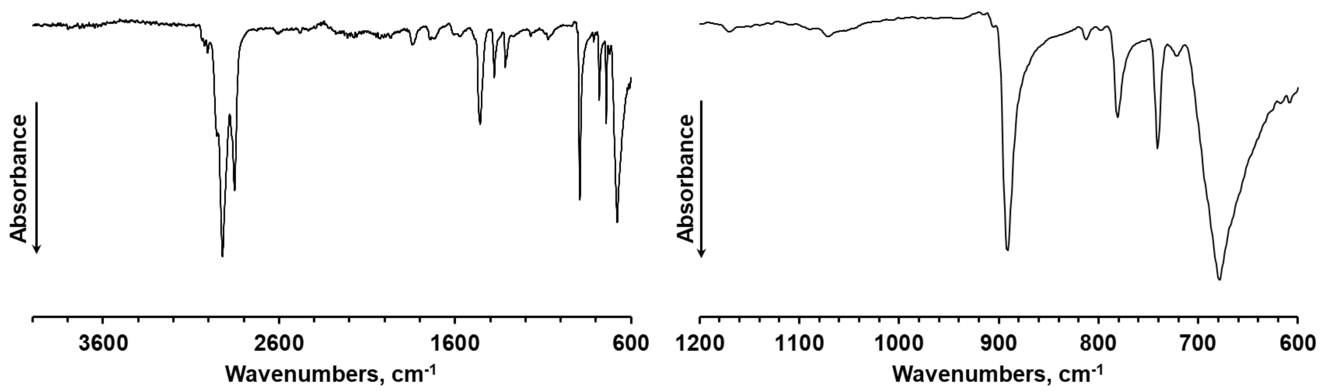

Figure S1. ATR-IR spectrum of **1**.

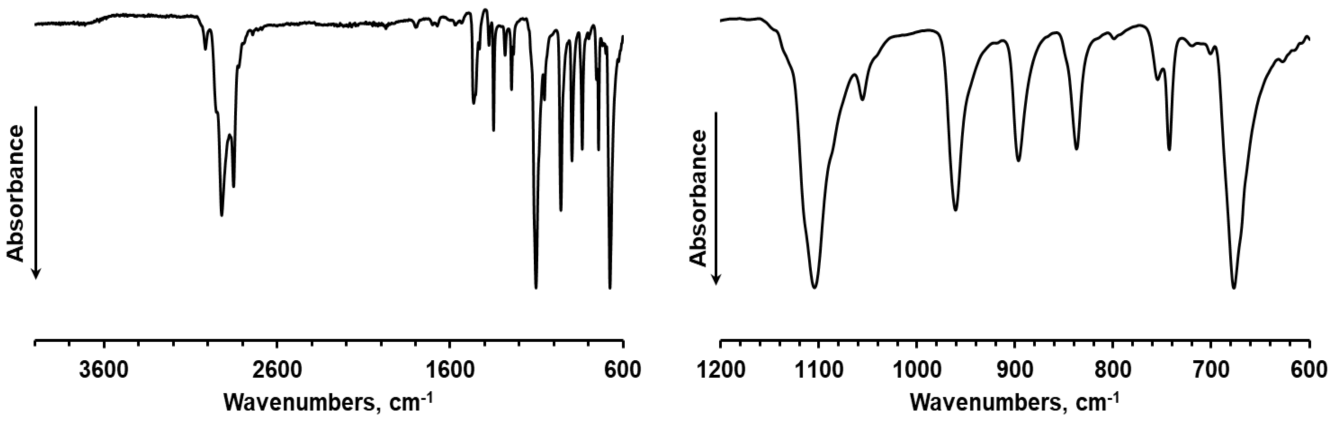

Figure S2. ATR-IR spectrum of **2**.

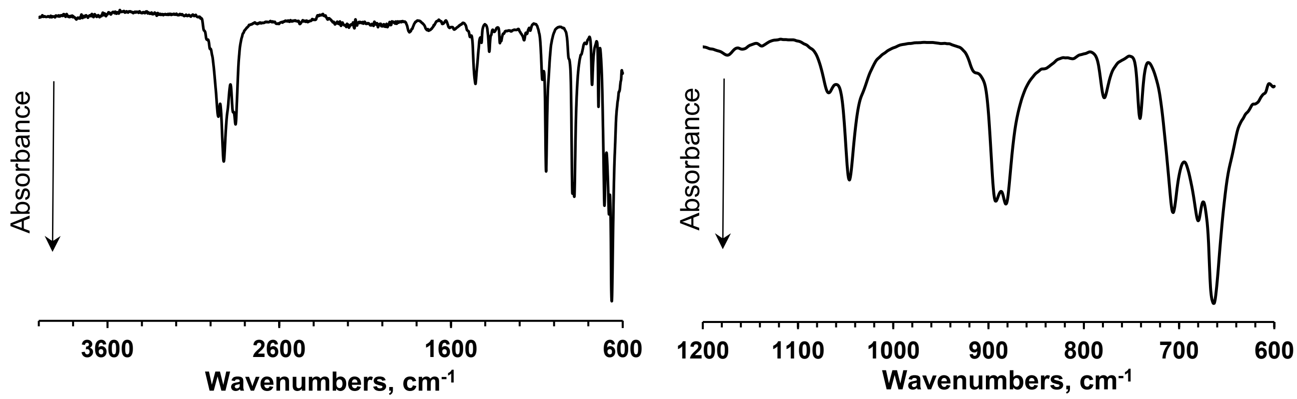

Figure S3. ATR-IR spectrum of **3**.

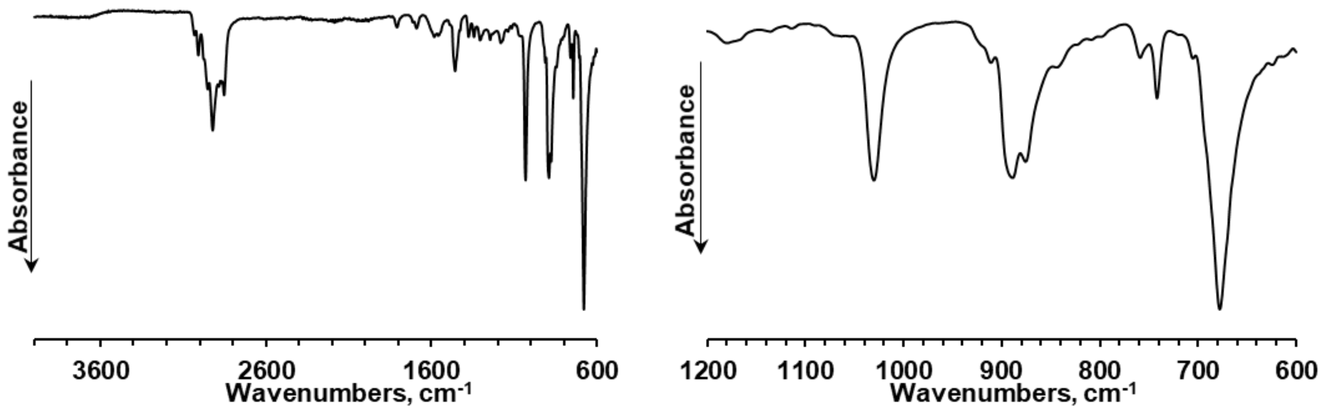

Figure S4. ATR-IR spectrum of **4**.

II. X-Ray Powder Diffraction and Le Bail Fit

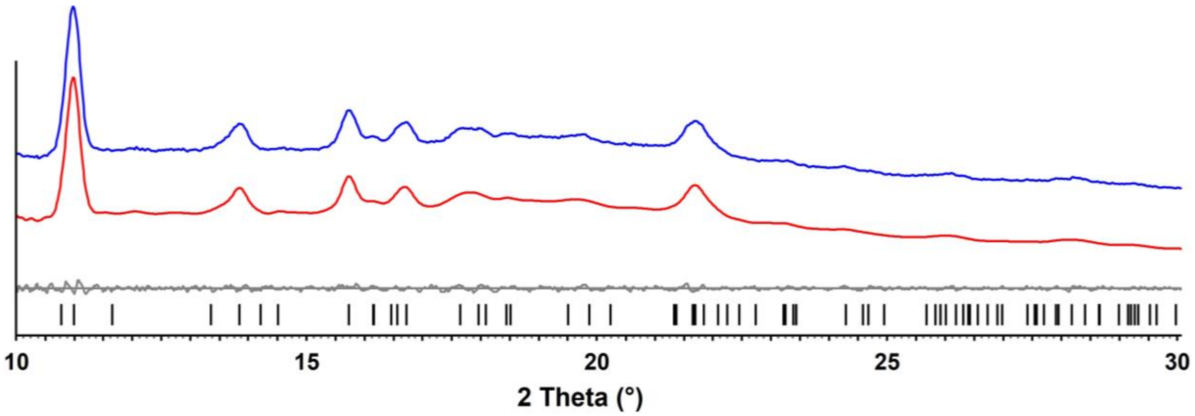

Figure S5. X-ray powder diffraction pattern of **1** and Le Bail fit. The blue and red lines are experimental and calculated patterns, respectively. The grey line is the difference curve with theoretical positions shown at the bottom in black.

Table S1. Comparison of unit cell parameters for **1** from single crystal data and the Le Bail fit.

**1**		
	Single crystal data (100 K)	Le Bail fit data (100 K)
Space Group	*P*2/*c*	
a (Å)	12.6009(16)	12.6027(18)
b (Å)	10.7948(19)	10.8101(16)
c (Å)	13.563(2)	13.5652(15)
α (°)	90.00	90.00
β (°)	101.972(2)	109.969(18)
γ (°)	90.00	90.00
V (Å^3)	1804.8(5)	1806.2(2)

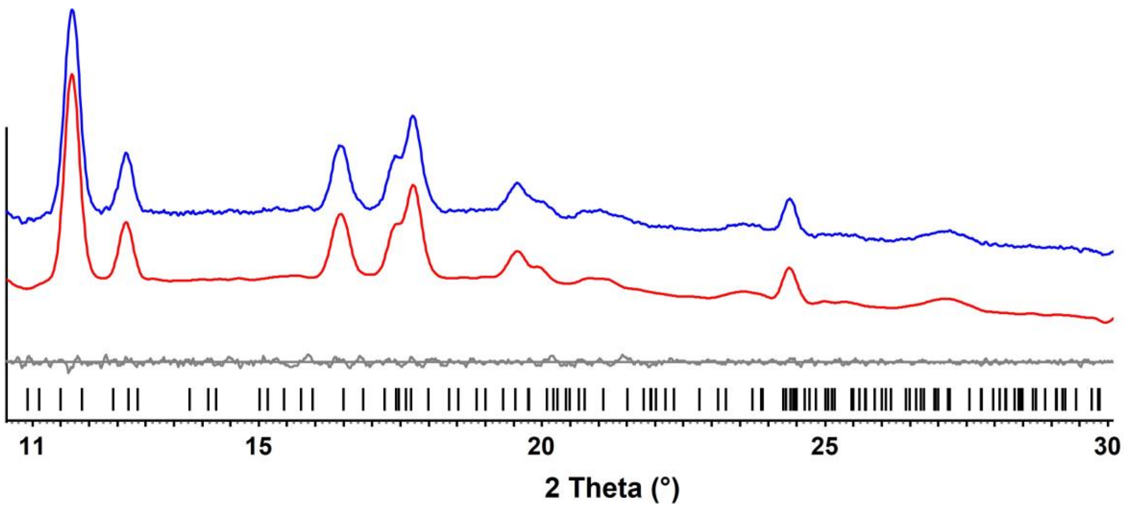

Figure S6. X-ray powder diffraction pattern of **2** and Le Bail fit. The blue and red lines are experimental and calculated patterns, respectively. The grey line is the difference curve with theoretical positions shown at the bottom in black.

Table S2. Comparison of unit cell parameters for **2** from single crystal data and the Le Bail fit.

**2**		
	Single crystal data (100 K)	Le Bail fit data (100 K)
Space Group	$P2_1/c$	
a (Å)	11.6156(13)	11.5985(16)
b (Å)	15.9533(18)	15.9901(20)
c (Å)	19.549(2)	19.5433(19)
α (°)	90.00	90.00
β (°)	90.079(2)	90.0784(19)
γ (°)	90.00	90
V (Å^3)	3622.6(7)	3626.8(3)

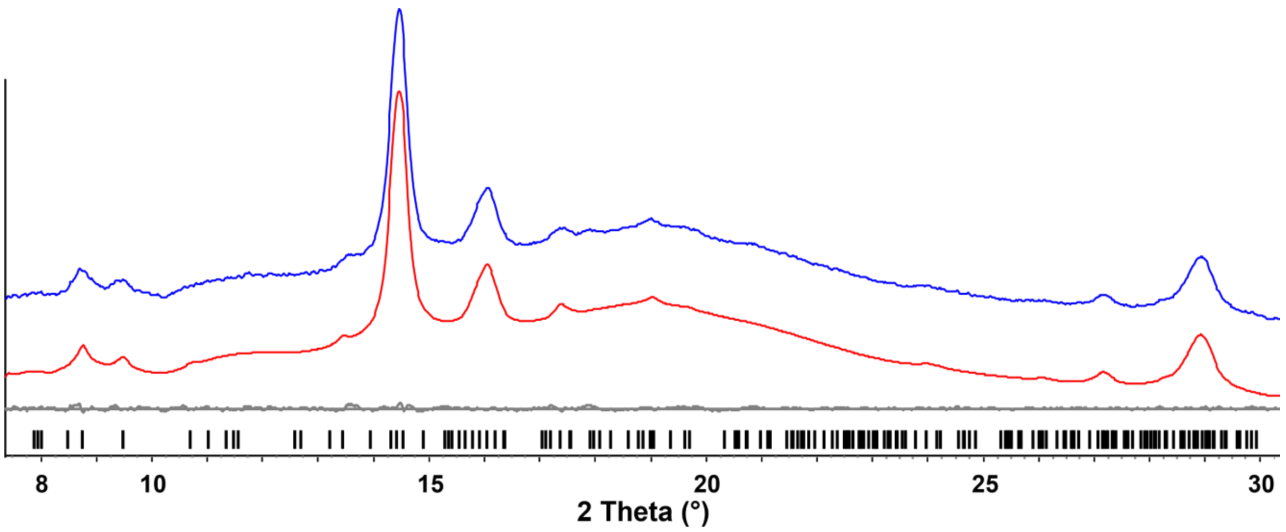

Figure S7. X-ray powder diffraction pattern of **3** and Le Bail fit. The blue and red lines are experimental and calculated patterns, respectively. The grey line is the difference curve with theoretical positions shown at the bottom in black.

Table S3. Comparison of unit cell parameters for **3** from single crystal data and the Le Bail fit.

**3**		
	Single crystal data (100 K)	Le Bail fit data (100 K)
Space Group	*P*-1	
a (Å)	12.3798(18)	12.3841(11)
b (Å)	12.9030(19)	12.8991(11)
c (Å)	16.6040(20)	16.5989(12)
α (°)	107.305(2)	107.315(13)
β (°)	98.137(2)	98.1295(9)
γ (°)	111.995(2)	111.991(13)
V (Å^3)	2250.0(6)	2249.4(2)

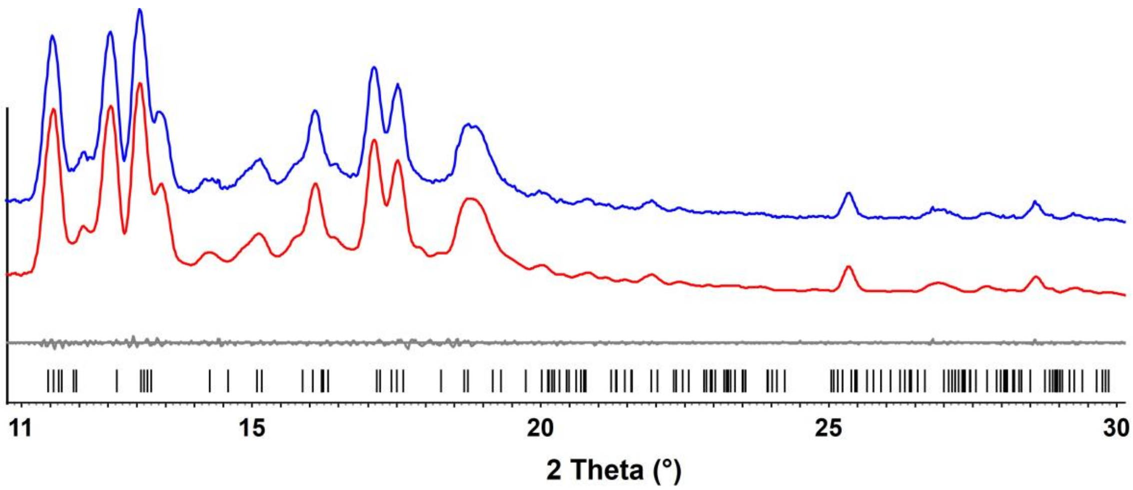

Figure S8. X-ray powder diffraction pattern of **4** and Le Bail fit. The blue and red lines are experimental and calculated patterns, respectively. The grey line is the difference curve with theoretical positions shown at the bottom in black.

Table S4. Comparison of unit cell parameters for **4** from single crystal data and the Le Bail fit.

	**4**	
	Single crystal data (100 K)	Le Bail fit data (100 K)
Space Group	$P2_1/c$	
a (Å)	9.2730(7)	9.2711(8)
b (Å)	13.9700(11)	13.9756(9)
c (Å)	26.160(2)	26.1582(10)
α (°)	90	90.00
β (°)	90.4780(10)	90.4804(9)
γ (°)	90	90
V (Å^3)	3388.8(5)	3387.8(11)

III. ^{1}H NMR Spectroscopic Investigation

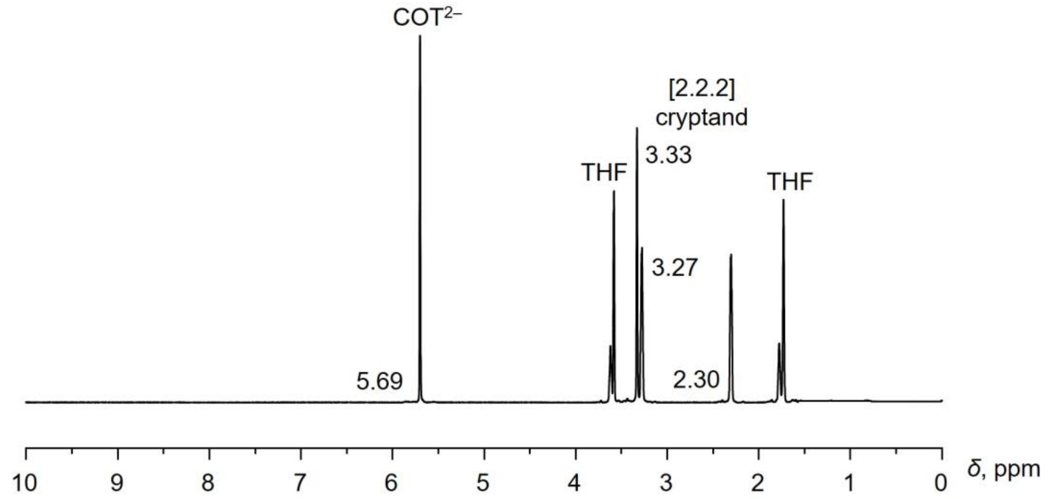

Figure S9. ^{1}H NMR spectrum of **1** in THF-d_8 at 25 °C.

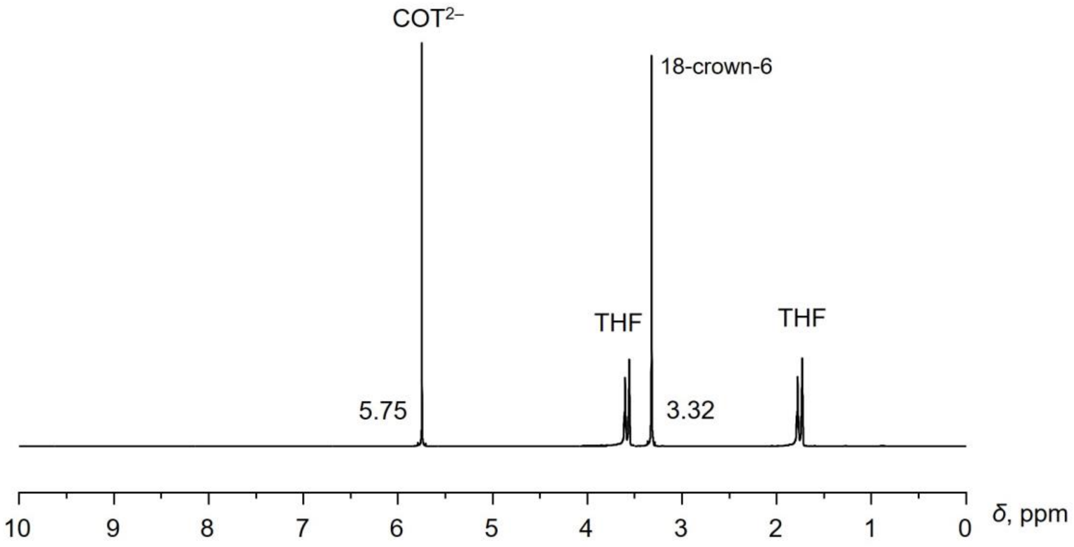

Figure S10. ^{1}H NMR spectrum of **2** in THF-d_8 at 25 °C.

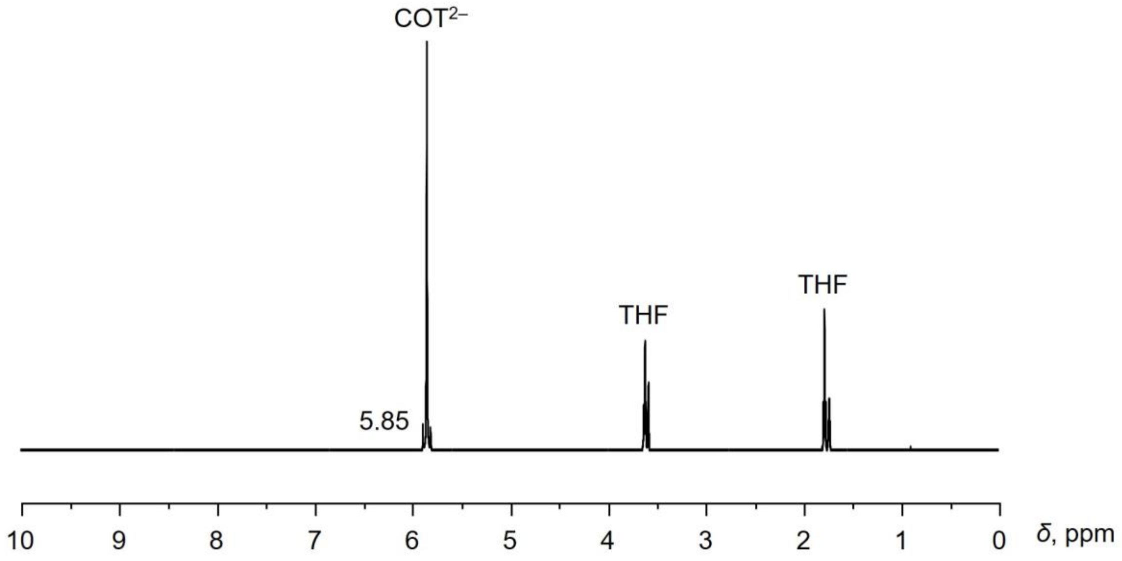

Figure S11. ^{1}H NMR spectrum of **3** in THF-d_8 at 25 °C.

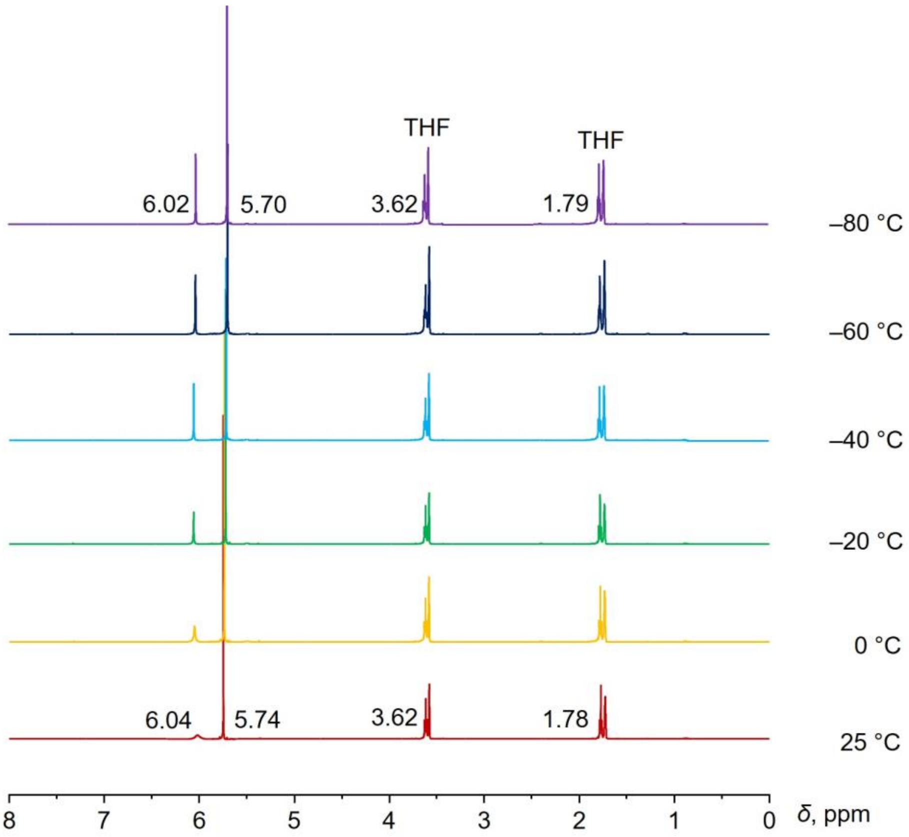

Figure S12. Variable-temperature ^{1}H NMR spectra of **4** in THF-d_8.

IV. ^{13}C NMR Spectroscopic Investigation

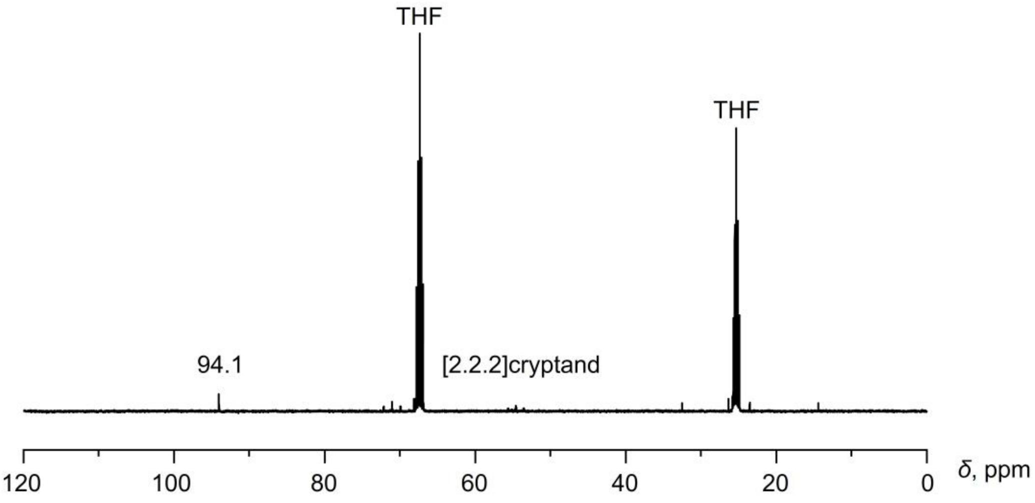

Figure S13. ^{13}C NMR spectrum of **1** in THF-d_8 at 25 °C.

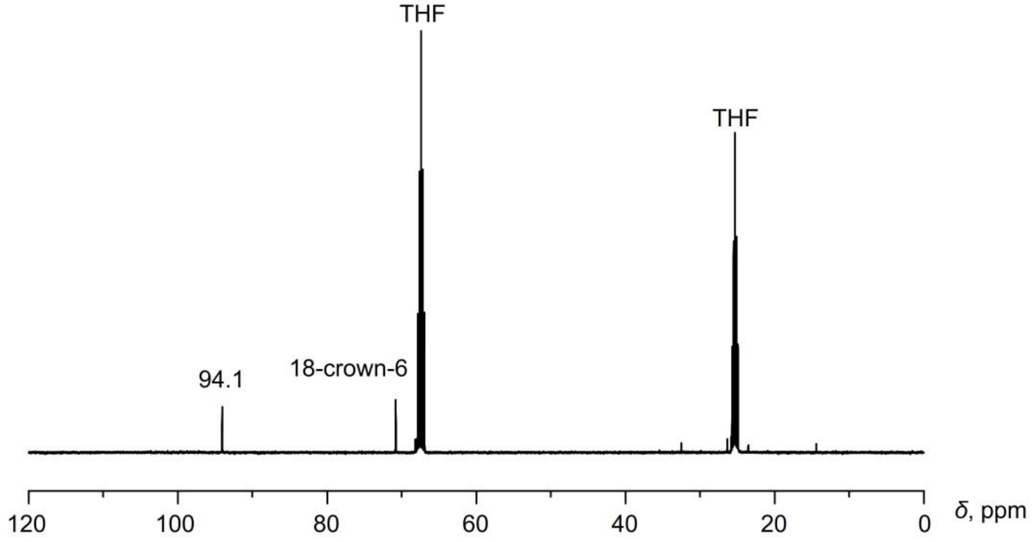

Figure S14. ^{13}C NMR spectrum of **2** in THF-d_8 at 25 °C.

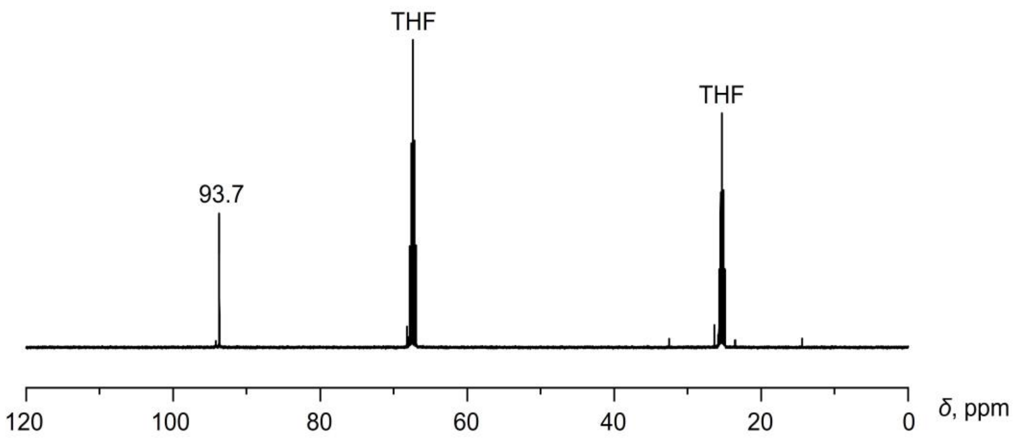

Figure S15. ^{13}C NMR spectrum of **3** in THF-d_8 at 25 °C.

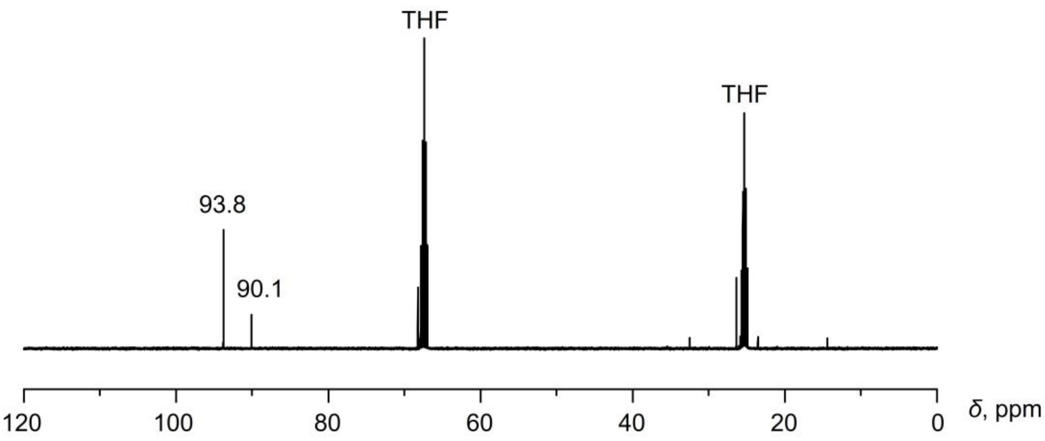

Figure S16. ^{13}C NMR spectrum of **4** in THF-d_8 at 25 °C.

V. ^{89}Y NMR Spectroscopic Investigation

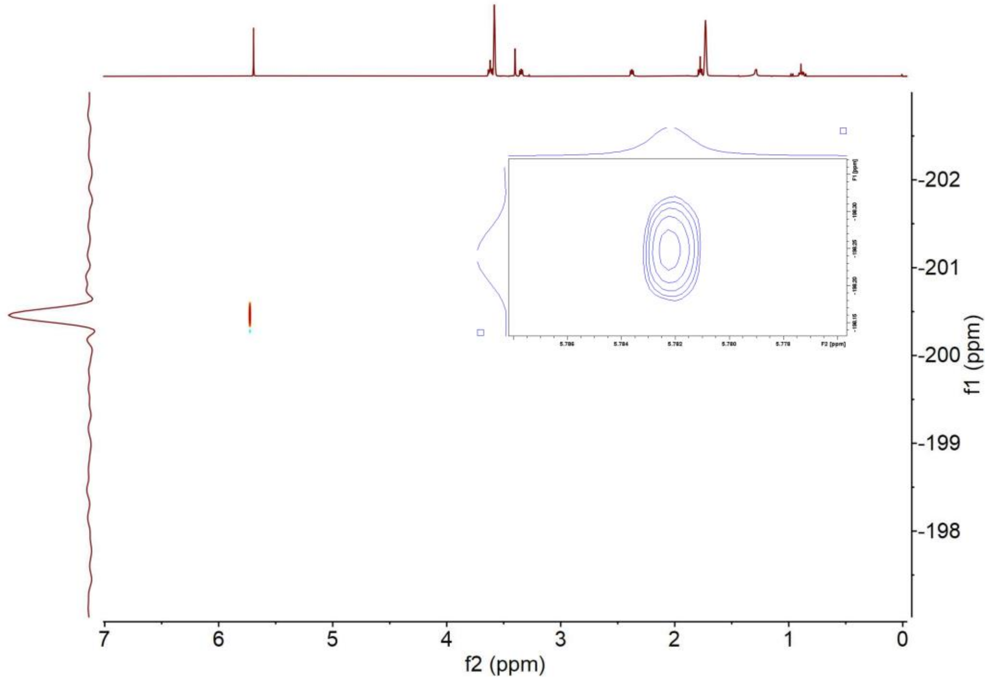

Figure S17. ^{1}H-^{89}Y HMQC spectrum of **1** in THF-d_8 at 25 °C.

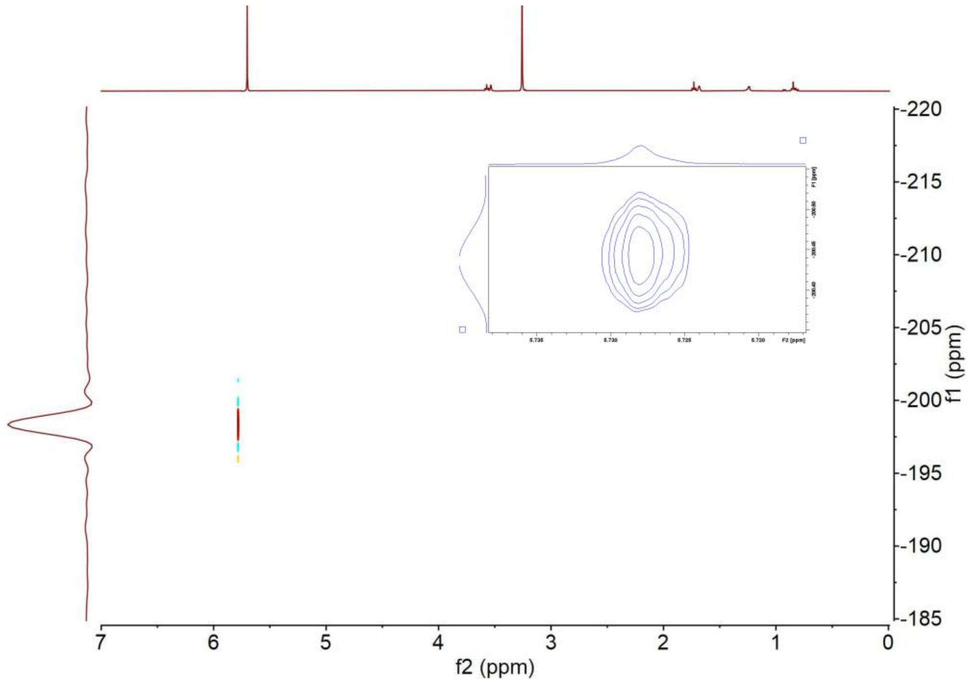

Figure S18. ^{1}H-^{89}Y HMQC spectrum of **2** in THF-d_8 at 25 °C.

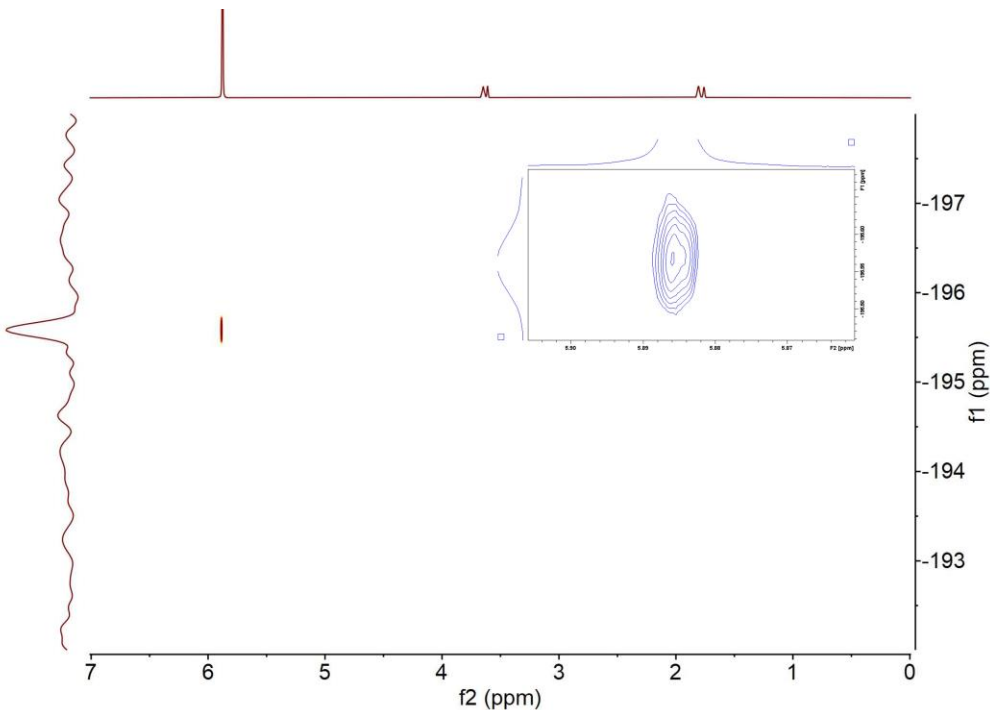

Figure S19. ^{1}H-^{89}Y HMQC spectrum of **3** in THF-d_8 at 25 °C.

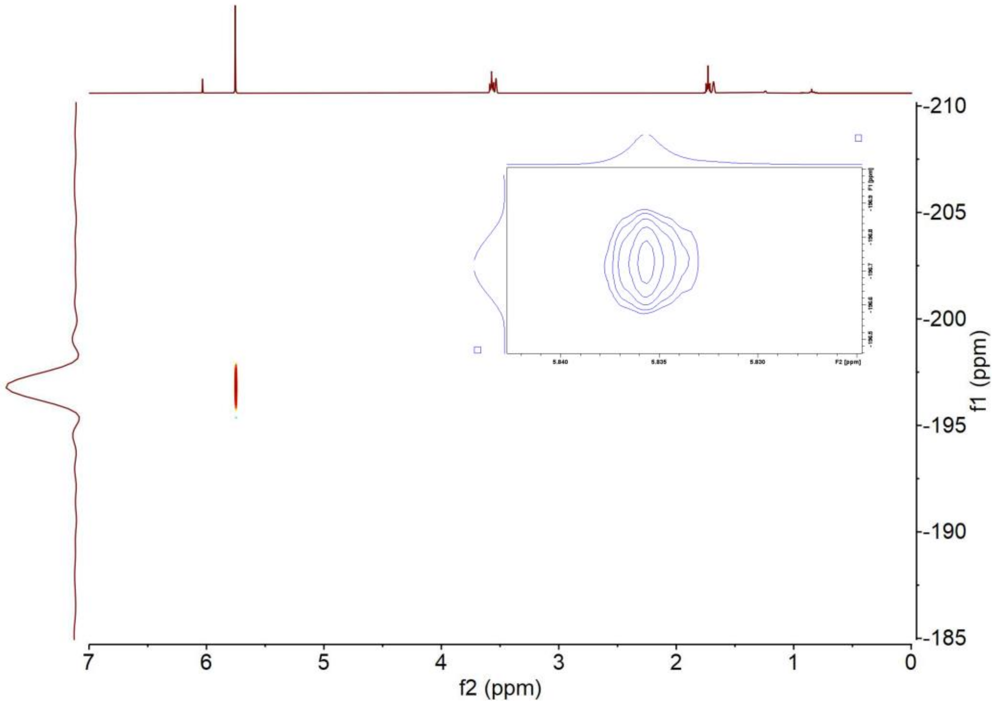

Figure S20. ^{1}H-^{89}Y HMQC spectrum of **4** in THF-d_8 at 25 °C.

VI. ^{13}C Solid-State NMR Spectroscopic Investigation

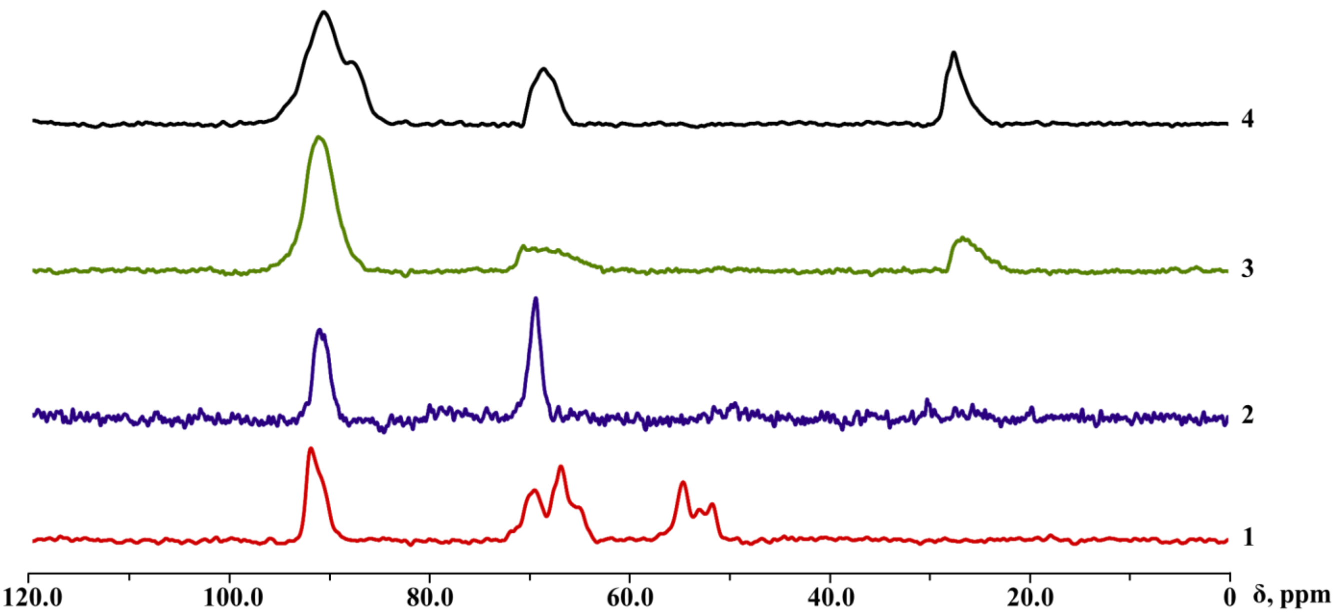

Figure S21. Solid-state ^{13}C CPMAS NMR spectra of [K([2.2.2]cryptand)][Y(COT)$_2$]) (**1**), [K(18-crown-6)(THF)$_2$][Y(COT)$_2$] (**2**), [Y_2K_2(COT)$_4$(THF)$_4$] (**3**), and [YKCa(COT)$_3$(THF)$_3$] (**4**).

VII. Diffusion Ordered Spectroscopy (DOSY) NMR

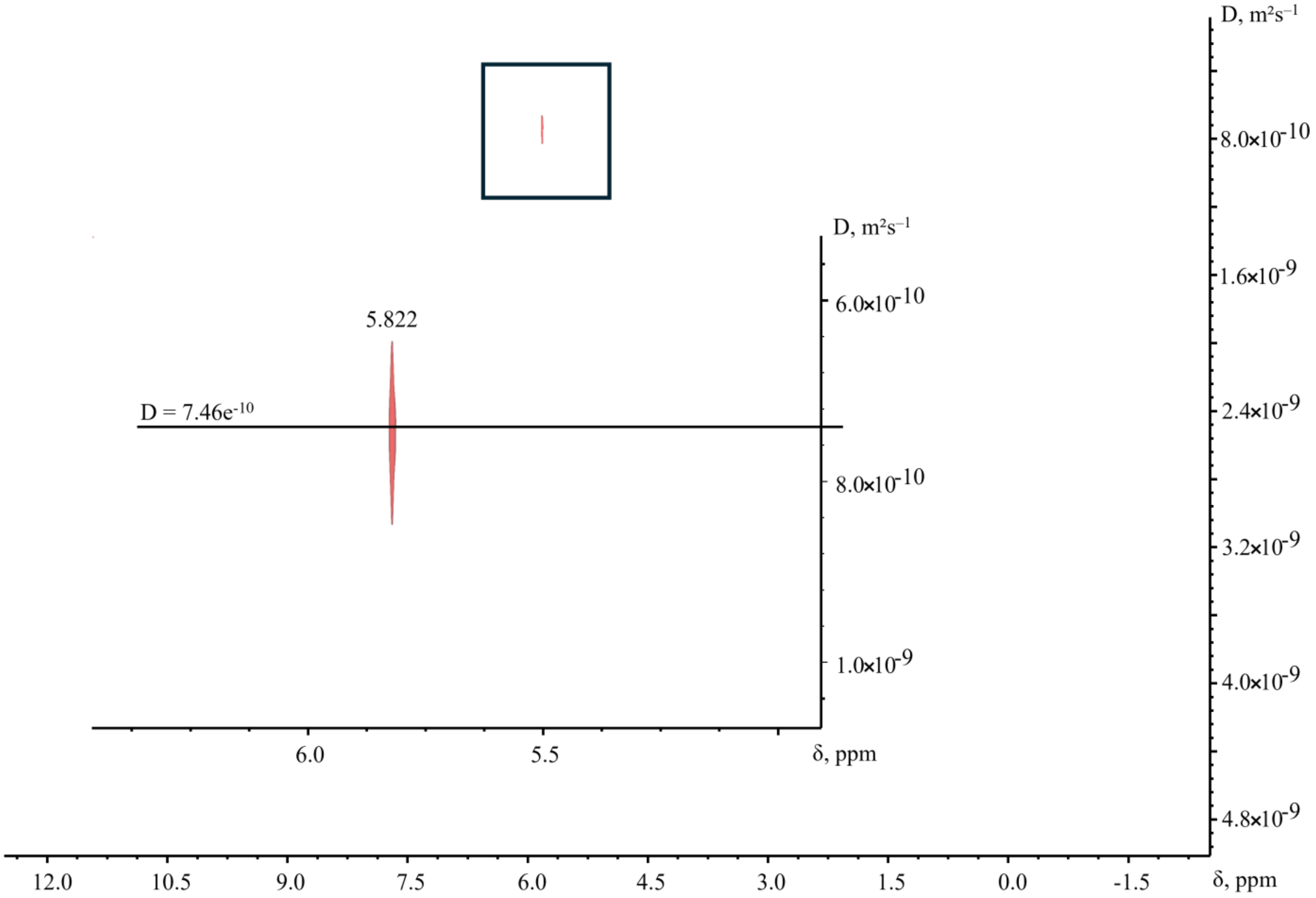

Figure S22. ^{1}H DOSY NMR of $Y_2K_2(COT)_4(THF)_4$ in THF-d_8 with diffusion coefficient.[1]

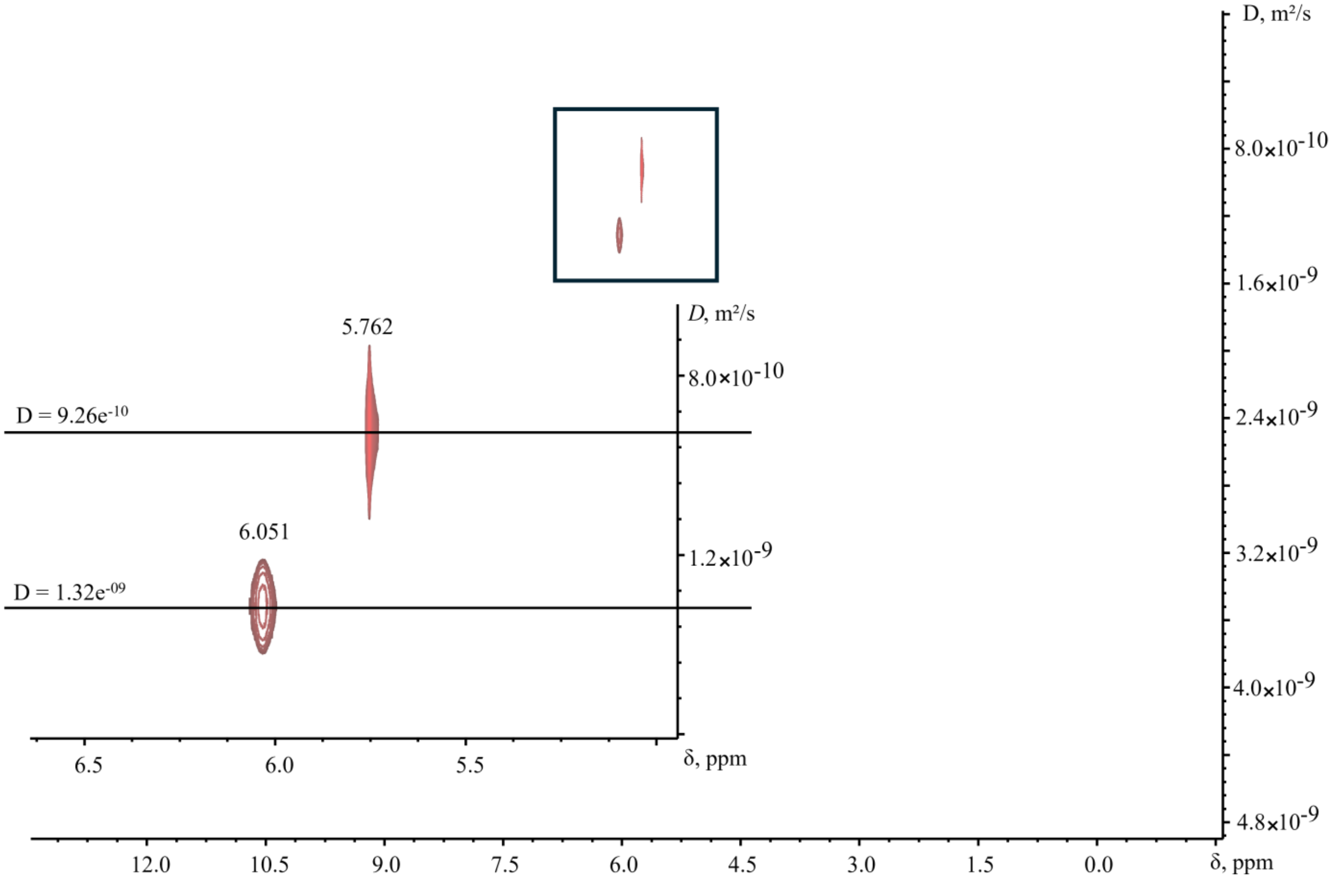

Figure S23. ^{1}H DOSY NMR of $YKCa(COT)_3(THF)_3$ in THF-d_8 showing fragments and diffusion coefficients.[1]

VIII. Crystal Structure Solution and Refinement Details

Data collections of **1**–**4** were performed on a Bruker D8 VENTURE X-ray diffractometer equipped with a PHOTON 100 CMOS shutterless mode detector and a Mo-target X-ray tube (λ = 0.71073 Å) at T = 100(2) K. Data reduction and integration were performed with the Bruker software package SAINT (version 8.38A).[2] Data were corrected for absorption effects using the empirical methods as implemented in SADABS (version 2016/2).[3] All structures were solved by SHELXT(version 2018/2)[4] and refined by full-matrix least-squares procedures using the Bruker SHELXTL (version 2018/3)[5] software package through the OLEX2 graphical interface.[6] All non-hydrogen atoms, including those in disordered parts, were refined anisotropically. The H atoms were also included at calculated positions and refined as riders, with U_{iso}(H) = 1.2 U_{eq}(C). In **2**·THF, one THF molecule was found to be disordered. All disordered molecules were modeled with two orientations with their relative occupancies refined. The geometries of the disordered parts were restrained to be similar. The anisotropic displacement parameters of the disordered molecules in the direction of the bonds were restrained to be equal with a standard uncertainty of 0.004 Å^2. They were also restrained to have the same U_{ij} components, with a standard uncertainty of 0.01 Å^2. The structure of **2**·THF was refined as a two-component inversion twin with twin law [-1 0 0 0 -1 0 0 0 1] with BASF parameter refined to 0.4578(10). Crystallographic data for **1**–**4** and details of the data collection and structure refinement are listed in Table S5. The ORTEP drawings, solid-state packings, and additional tables with the key bond distances and angles, are shown below.

Table S5. Crystal data and structure refinement parameters for **1–4**.

Compound	**1**	**2·THF**	**3**	**4**
Empirical formula	$C_{34}H_{52}KYO_6N_2$	$C_{36}H_{56}KYO_8$	$C_{48}H_{64}K_2Y_2O_4$	$C_{36}H_{48}CaKYO_3$
Formula weight	712.78	744.81	961.01	696.83
Temperature (K)	100(2)	100(2)	100(2)	100(2)
Wavelength (Å)	0.71073	0.71073	0.71073	0.71073
Crystal system	Monoclinic	Monoclinic	Triclinic	Monoclinic
Space group	$P2/c$	$P2_1/c$	P–1	$P2_1/c$
a (Å)	12.6009(16)	11.6156(13)	12.3798(18)	9.2730(7)
b (Å)	10.7948(19)	15.9533(18)	12.9030(19)	13.9700(11)
c (Å)	13.563(2)	19.549(2)	16.604(2)	26.160(2)
α (°)	90.00	90.00	107.305(2)	90.00
β (°)	101.972(2)	90.079(2)	98.137(2)	90.4780(10)
γ (°)	90.00	90.00	111.995(2)	90.00
V (Å^3)	1804.8(5)	3622.6(7)	2250.0(6)	3388.8(5)
Z	2	4	2	4
ρ_{calcd} (g·cm^{-3})	1.312	1.366	1.418	1.366
μ (mm^{-1})	1.774	1.773	2.793	2.028
F(000)	752	1576	1000	1464
Crystal size (mm)	0.04×0.05×0.16	0.03×0.06×0.12	0.05×0.18×0.26	0.02×0.08×0.52
θ range for data collection (°)	3.01-25.00	3.01-28.36	2.89-30.57	3.05-33.19
Reflections collected	30277	73724	57025	80545
Independent reflections	3178 [R_{int} = 0.0937]	9028 [R_{int} = 0.0789]	13463 [R_{int} = 0.0503]	12941 [R_{int} = 0.0668]
Transmission factors (min/max)	0.5987/0.7054	0.6295/0.7249	0.4918/0.7461	0.5158/0.7465
Data/restraints/params.	3178/0/201	9028/220/462	13463/0/505	12941/0/379
$R1$,[a] $wR2$[b] ($I > 2\sigma(I)$)	0.0754, 0.1283	0.0378, 0.0663	0.0506, 0.1007	0.0385, 0.0651
$R1$,[a] $wR2$[b] (all data)	0.1273, 0.1463	0.0633, 0.0748	0.0729, 0.1087	0.0665, 0.0721
Quality-of-fit[c]	1.125	1.026	1.080	1.018

[a] $R_1 = \Sigma||F_o|-|F_c||/\Sigma|F_o|$. [b] $wR_2 = [\Sigma[w(F_o^2-F_c^2)^2]/\Sigma[w(F_o^2)^2]]$.
[c] Quality-of-fit $S = [\Sigma[w(F_o^2-F_c^2)^2]/(N_{obs}-N_{params})]^{½}$, based on all data.

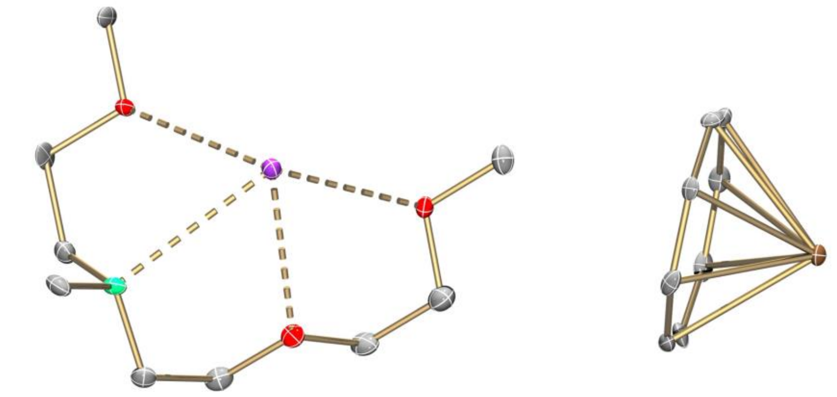

Figure S24. ORTEP drawing of the asymmetric unit of **1**, drawn with thermal ellipsoids at the 40% probability level. All hydrogen atoms are removed for clarity. The color scheme used: C grey, O red, N spring green, K dark orchid, Y brown.

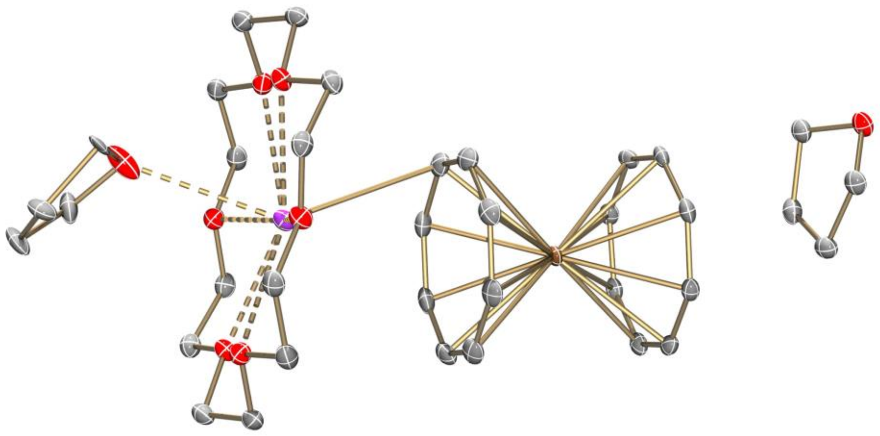

Figure S25. ORTEP drawing of the asymmetric unit of **2**·THF, drawn with thermal ellipsoids at the 40% probability level. All hydrogen atoms are removed for clarity. The color scheme used: C grey, O red, K dark orchid, Y brown.

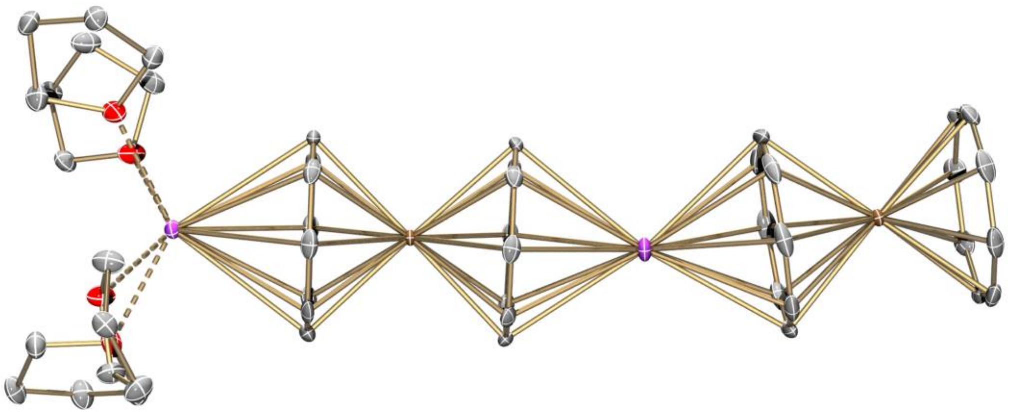

Figure S26. ORTEP drawing of the asymmetric unit of **3**, drawn with thermal ellipsoids at the 40% probability level. All hydrogen atoms are removed for clarity. The color scheme used: C grey, O red, K dark orchid, Y brown.

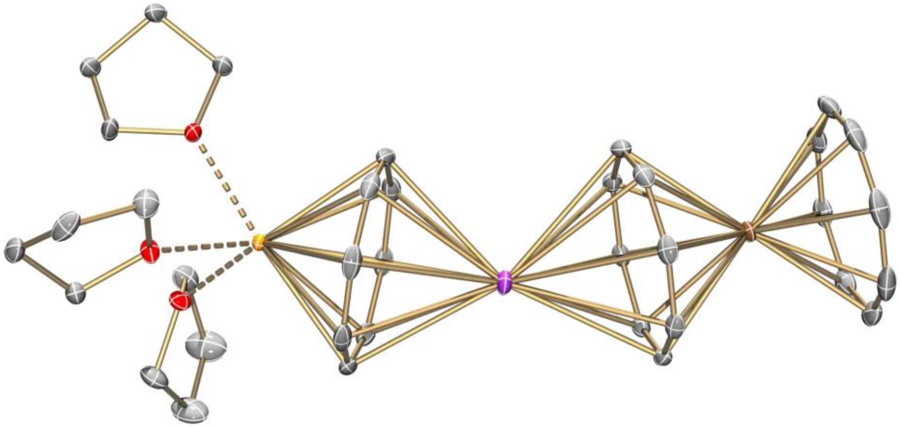

Figure S27. ORTEP drawing of the asymmetric unit of **4**, drawn with thermal ellipsoids at the 40% probability level. All hydrogen atoms are removed for clarity. The color scheme used: C grey, O red, K dark orchid, Ca orange, Y brown.

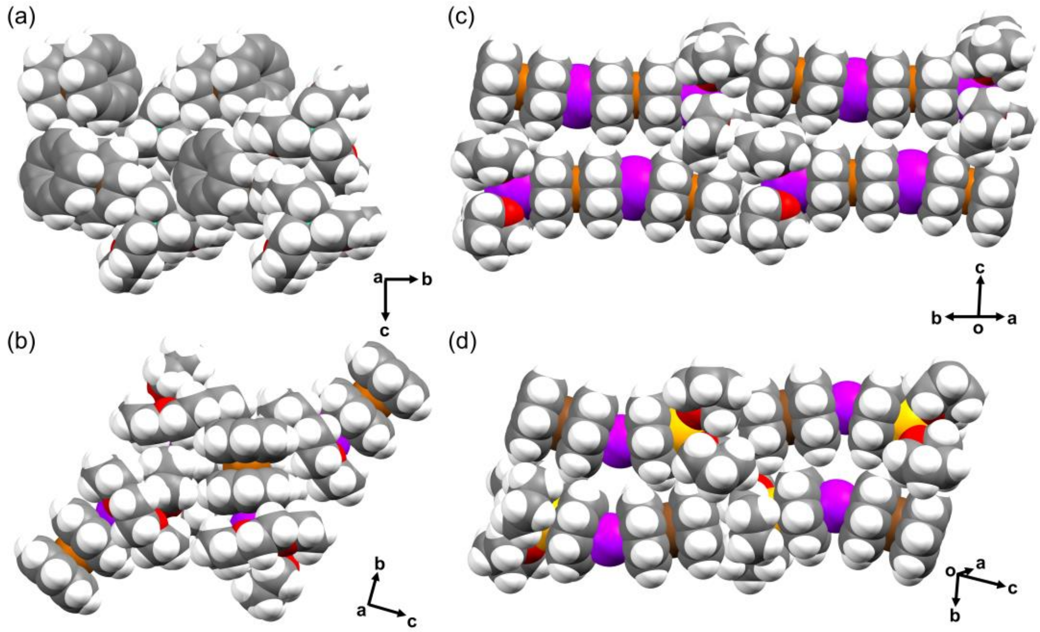

Figure S28. Solid-state packing of **1**–**4** in space-filling models.

Table S6. Selected bond length distances (Å) in **1**–**4**, along with labeling schemes.

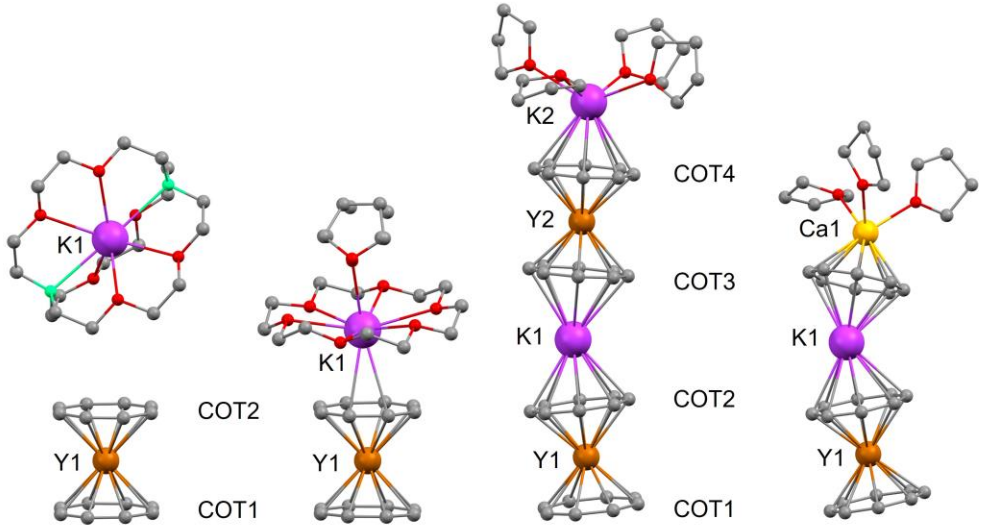

	**1**	**2**	**3**	**4**
Y1–$C_{COT1,\,avg}$	2.637(6)	2.631(4)	2.616(3)	2.615(2)
Y1 to $centroid_{COT1}$	1.884(6)	1.883(4)	1.857(3)	1.859(2)
Y1–$C_{COT2,\,avg}$	2.637(6)	2.638(4)	2.684(3)	2.651(2)
Y1 to $centroid_{COT2}$	1.884(6)	1.892(4)	1.952(3)	1.907(2)
K1–$C_{COT2,\,avg}$	–	3.231(2)/3.310(4)	3.088(3)	3.086(2)
K1 to $centroid_{COT2}$	–	3.198(4)	2.479(3)	2.476(2)
K1–$C_{COT3,\,avg}$	–	–	3.125(3)	3.030(2)
K1 to $centroid_{COT3}$	–	–	2.521(3)	2.407(2)
Y2–$C_{COT3,\,avg}$	–	–	2.648(3)	–
Y2 to $centroid_{COT3}$	–	–	1.897(3)	–
Y2–$C_{COT4,\,avg}$	–	–	2.638(3)	–
Y2 to $centroid_{COT4}$	–	–	1.883(3)	–
K2–$C_{COT4,\,avg}$	–	–	3.189(3)	–
K2 to $centroid_{COT4}$	–	–	2.601(3)	–
Ca1–$C_{COT3,\,avg}$	–	–	–	2.656(2)
Ca1 to $centroid_{COT3}$	–	–	–	1.914(2)

Table S7. Selected dihedral angles (°) in **1**–**4**.

	**1**	**2**	**3**	**4**
COT1/COT2	0	2.2	3.0	0.8
COT2/COT3	–	–	11.6	27.5
COT3/COT4	–	–	2.2	–

IX. Calculation Details

All geometry optimization and energy calculations were performed with ORCA 5.0.4.[7] The geometries of complexes were optimized using PBE0[8-9] functional in conjunction with def2-TZVP basis set[10] for Y atoms and def2-SVP basis set[10] for other atoms. Dispersion effects were accounted for using Grimme's D3 correction with Becke−Johnson damping.[11-12] The geometries of the initial model were extracted from crystal structure data of **1**–**4**, and the interstitial THF molecule in **2** was removed. TD-DFT was calculated using B3LYP[13-14] functional in conjunction with def2-TZVP[10] basis set and conductor-like polarizable continuum model (CPCM) of THF. It should be noted that optimization with PBE0/def2-TZVP(Y)/def2-SVP(C,H,O,K), PBE0/def2-TZVP, B3LYP/def2-TZVP(Y)/def2-SVP(C,H,O,K), B3LYP/def2-TZVP were compared but no significant difference was found, so optimized geometries of PBE0/def2-TZVP(Y)/def2-SVP(C,H,O,K) were used. Similarly, TD-DFT calculations with B3LYP/def2-TZVP were used. Additionally, the geometry and TD-DFT of the anionic $[Y(COT)_2]^-$ sandwich was calculated for comparison with title complexes.

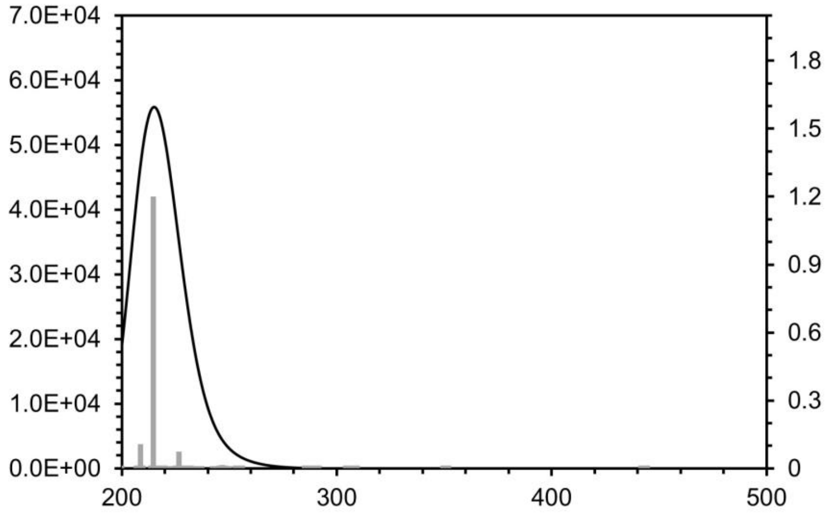

Figure S29. The calculated UV-vis spectrum of $[Y(COT)_2]^-$ (in nm) at the B3LYP-D3BJ/def2-TZVP level of theory.

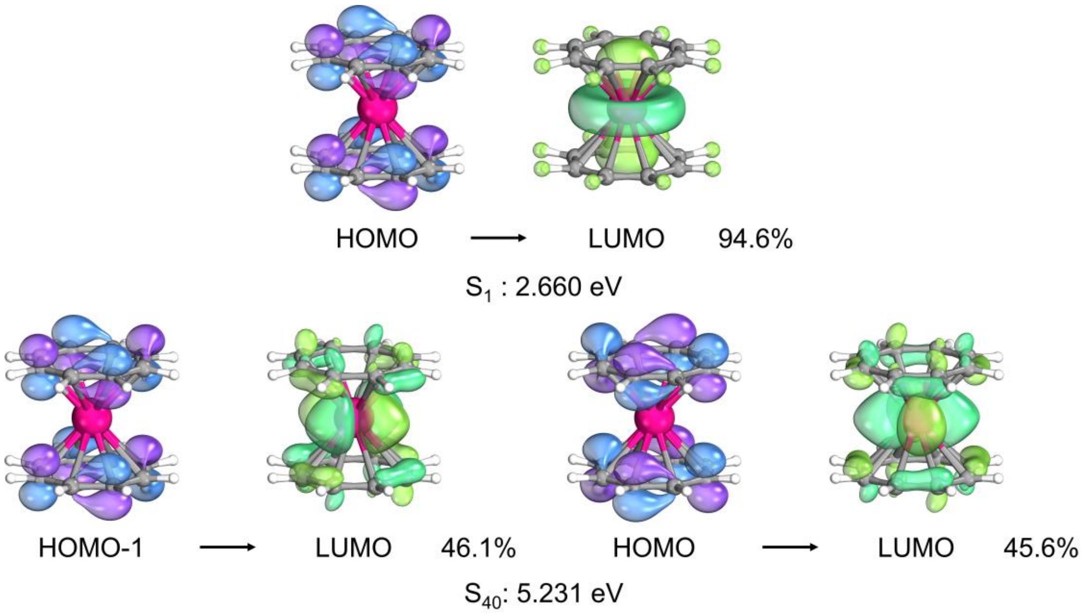

Figure S30. Selected major electronic transitions in $[Y(COT)_2]^-$.

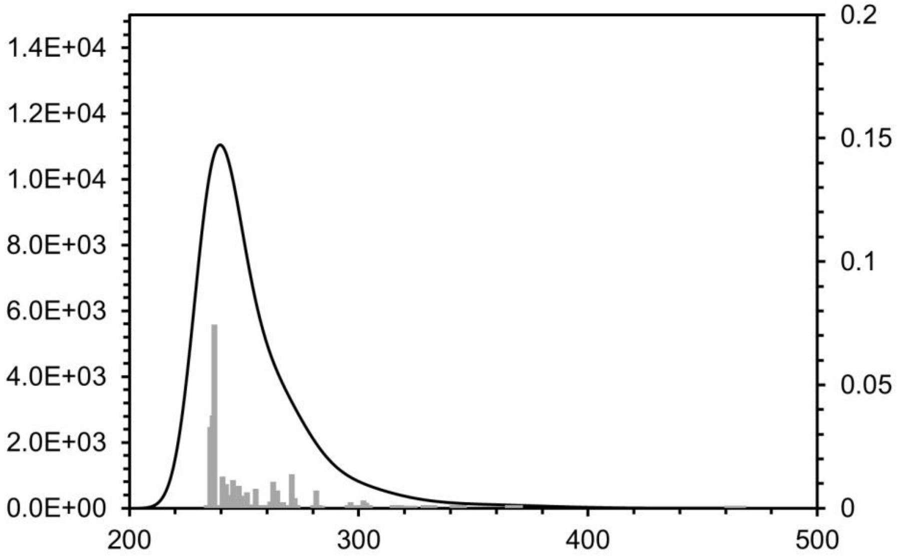

Figure S31. The calculated UV-vis spectrum of **1** (in nm) at the B3LYP-D3BJ/def2-TZVP level of theory.

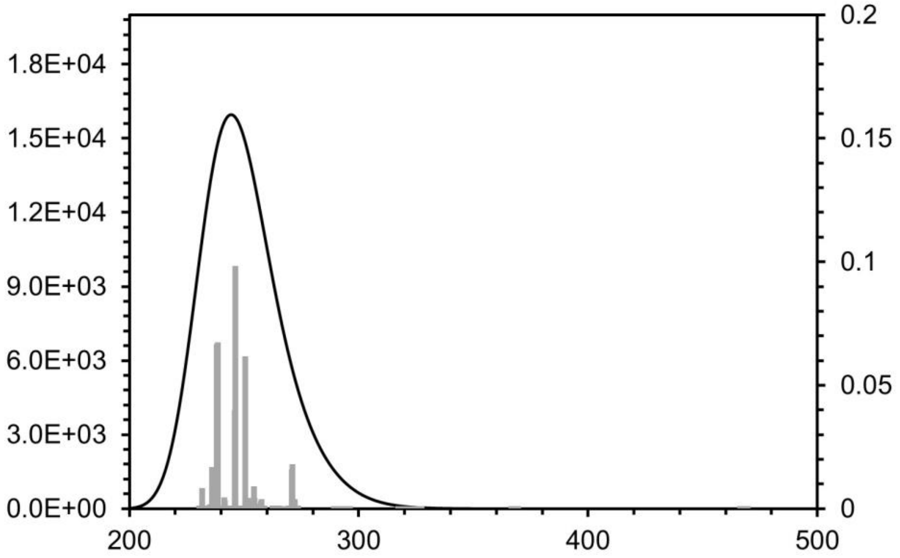

Figure S32. The calculated UV-vis spectrum of **2** (in nm) at the B3LYP-D3BJ/def2-TZVP level of theory.

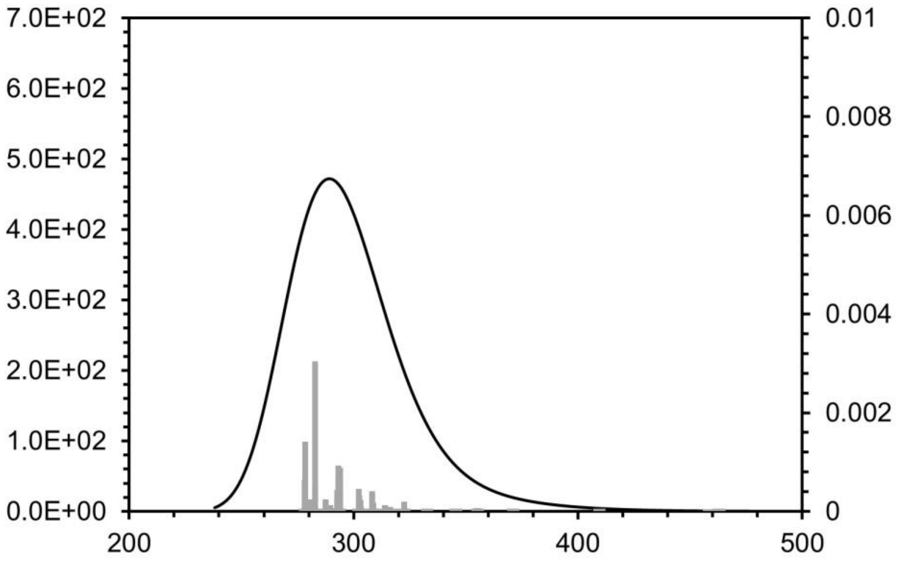

Figure S33. The calculated UV-vis spectrum of **3** (in nm) at the B3LYP-D3BJ/def2-TZVP level of theory.

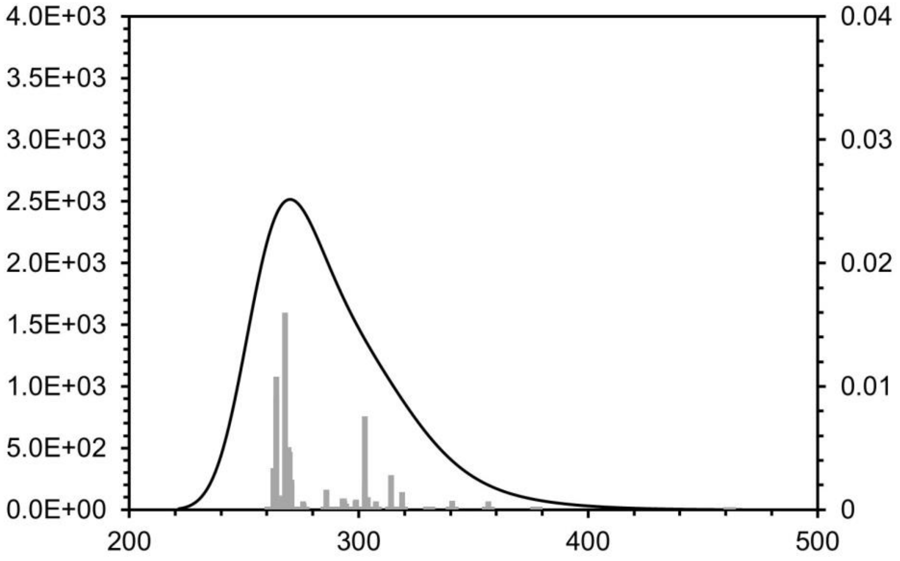

Figure S34. The calculated UV-vis spectrum of **4** (in nm) at the B3LYP-D3BJ/def2-TZVP level of theory.

X. References

[1] Stejskal, E.O.; Tanner, J.E. Spin diffusion Measurements: Spin Echoes in the Presence of a Time-Dependent Field Gradient. *J. Chem. Phys.* **1965**, *42*, 288-292.

[2] SAINT; part of Bruker APEX3 software package (version 2017.3-0): Bruker AXS, **2017**.

[3] SADABS; part of Bruker APEX3 software package (version 2017.3-0): Bruker AXS, **2017**.

[4] G. M. Sheldrick, *Acta Crystallogr.* **2015**, *A71*, 3-8.

[5] G. M. Sheldrick, *Acta Crystallogr.* **2015**, *C71*, 3-8.

[6] O. V. Dolomanov, L. J. Bourhis, R. J. Gildea, J. A. K. Howard, H. Puschmann, *J. Appl. Crystallogr.* **2009**, *42*, 339–341.

[7] F. Neese, F. Wennmohs, U. Becker, C. Riplinger, *J. Chem. Phys.* The ORCA quantum chemistry program package, **2020**, *152*, 224108.

[8] Perdew, J. P.; Burke, K.; Ernzerhof, M. Generalized Gradient Approximation Made Simple. Phys. Rev. Lett. 1996, 77, 3865-3868.

[9] Perdew, J. P.; Burke, K.; Ernzerhof, M. Generalized Gradient Approximation Made Simple. Phys. Rev. Lett. 1997, 78, 1396-1396.

[10] F. Weigend, R. Ahlrichs, *Phys. Chem. Chem. Phys.* Balanced Basis Sets of Split Valence, Triple Zeta Valence and Quadruple Zeta Valence Quality for H to Rn: Design and Assessment of Accuracy, **2005**, *7*, 3297-3305.

[11] S. Grimme, J. Antony, S. Ehrlich, H. Krieg, *J. Chem. Phys.* A Consistent and Accurate *ab* Initio Parametrization of Density Functional Dispersion Correction (DFT-D) for the 94 Elements H-Pu, **2010**, *132*, 154104.

[12] S. Grimme, S. Ehrlich, L. Goerigk, *J. Comput. Chem.* Effect of the Damping Function in Dispersion Corrected Density Functional Theory, **2011**, *32*, 1456-1465.

[13] C. Lee, W. Yang, R. G. Parr, *Phys. Rev. B* Development of the Colle-Salvetti correlation-energy formula into a functional of the electron density, **1988**, *37*, 785-789.

[14] A. D. Becke, *J. Chem. Phys.* Density-functional thermochemistry. III. The role of exact exchange, **1993**, *98*, 5648-5652.
